# The antibacterial activity of a novel highly thermostable endolysin, LysKP213, against Gram-negative pathogens is enhanced when combined with outer membrane permeabilizing agents

**DOI:** 10.3389/fmicb.2024.1454618

**Published:** 2024-10-08

**Authors:** Dingjian Chu, Jing Lan, Lu Liang, Kaide Xia, Linlin Li, Lan Yang, Hongmei Liu, Tingting Zhang

**Affiliations:** ^1^Engineering Research Center of Health Medicine Biotechnology of Institution of Higher Education of Guizhou Province, School of Biology and Engineering (School of Modern Industry for Health and Medicine), Guizhou Medical University, Guiyang, China; ^2^Guiyang Maternal and Child Health Hospital, Guiyang, China; ^3^Shanghai Institute of Phage, Shanghai Public Health Clinical Center, Fudan University, Shanghai, China

**Keywords:** phage, phage endolysin, Gram-negative bacteria, polymyxin B, cecropin A, synergy

## Abstract

Phages and phage-encoded lytic enzymes are promising antimicrobial agents. In this study, we report the isolation and identification of bacteriophage KP2025 from *Klebsiella pneumoniae*. Bioinformatics analysis of KP2025 revealed a putative endolysin, LysKP213, containing a T4-like_lys domain. Purified LysKP213 was found to be highly thermostable, retaining approximately 44.4% of its lytic activity after 20 h of incubation at 95°C, and approximately 57.5% residual activity after 30 min at 121°C. Furthermore, when administered in combination with polymyxin B or fused at the N-terminus with the antimicrobial peptide cecropin A (CecA), LysKP213 exhibited increased antibacterial activity against Gram-negative pathogens, including *K. pneumoniae*, *Pseudomonas aeruginosa*, *Acinetobacter baumannii*, and *Escherichia coli*, both *in vitro* and *in vivo*. These results indicated that LysKP213 is a highly thermostable endolysin that, when combined with or fused with an outer membrane permeabilizer, has enhanced antibacterial activity and is a candidate agent for the control of infections by Gram-negative pathogens.

## Introduction

The emergence of multidrug-resistant (MDR) microorganisms has led to a serious health challenge worldwide due to the limitations of currently available antibiotics (Hu et al., 2021; Bharadwaj et al., 2022; Kocsis, 2023; Zhu et al., 2023). The World Health Organization (WHO) has published a list of “priority pathogens,” which includes *Klebsiella pneumoniae*, *Pseudomonas aeruginosa*, *Acinetobacter baumannii*, *Staphylococcus aureus*, and *Enterobacter* spp. (Tacconelli et al., 2018). Polymyxins (polymyxin B and colistin [polymyxin E]) are the main class of antibiotics currently used against MDR Gram-negative bacteria in the clinic (Roberts et al., 2015; Nang et al., 2021). However, the utility of polymyxins as “last-line antibiotics” is limited due to high rates of toxicity (Nang et al., 2021). Consequently, alternative antibacterial agents for the treatment of infections by MDR microorganisms are urgently required.

Endolysins are lytic enzymes produced by bacteriophages during the last step of their lifecycle to digest the peptidoglycan layer of the bacterial cell wall (Gondil et al., 2020; Abdelrahman et al., 2021). Endolysin has a low probability of developing resistance. Therefore, endolysins are considered promising alternatives to counter drug-resistant infections (Danis-Wlodarczyk et al., 2021; Khan et al., 2021; Liu B. et al., 2023; Liu et al., 2023a). In addition, in recent years, increasingly animal models have shown efficacy of endolysins against bacterial infections *in vivo* (Fursov et al., 2020; Schmelcher and Loessner, 2021). However, most endolysins are limited in their ability to degrade the peptidoglycan layer owing to the presence of an outer membrane (OM) in Gram-negative bacteria (Lai et al., 2020; Shah et al., 2023; Wang et al., 2024). Thus, numerous studies have focused on combining endolysins with an OM permeabilizer, such as EDTA (a chelating agent), organic acids, and colistin (Thummeepak et al., 2016; Blasco et al., 2020; Hong et al., 2022; Sitthisak et al., 2023). Despite its usefulness as an OM permeabilizer, EDTA inhibits blood clotting at low concentrations; thus, the potential applications of an endolysin-EDTA combination are limited to the topical treatment of localized bacterial infections. Combinations of different endolysins with colistin have been shown to have marked antibacterial activity against *A. baumannii* infection, resulting in lower doses of colistin and, consequently, reduced toxicity (Thummeepak et al., 2016; Blasco et al., 2020; Hong et al., 2022; Sitthisak et al., 2023). Additionally, several studies have recently highlighted the substantial antimicrobial activity of the combination of phage endolysins with antimicrobial peptides (AMPs) capable of permeabilizing the OM of Gram-negative bacteria (Abdelkader et al., 2022; Hong et al., 2022; Lim et al., 2022; Zhang et al., 2022; Xuan et al., 2023).

In this study, we report the isolation and identification of an endolysin, LysKP213, from the *K. pneumoniae* bacteriophage KP2025. LysKP213 displayed antimicrobial activity against a relatively broad spectrum of Gram-negative bacteria after EDTA treatment. In addition, when combined with polymyxin B or fused with the AMP cecropin A (CecA), LysKP213 exhibited enhanced antibacterial activity against the tested strains both *in vitro* and *in vivo* relative to that seen with polymyxin B or CecA alone. These results highlight the potential of endolysin LysKP213 as a candidate antibacterial agent for the control of infections with Gram-negative pathogens.

## Materials and methods

### Bacterial strains and growth conditions

The tested bacteria were obtained from clinical samples (Supplementary Tables S1, S4). The bacteria were mixed with Luria-Bertani (LB) broth, cultured at 37°C with shaking and stored at −80°C in 50% glycerol (Wei et al., 2023).

### Bacteriophage isolation

Hospital sewage was centrifuged at 8,000 rpm for 10 min, filtered through a 0.22-μm filter, and stored at 4°C for later use. Bacteriophage isolation was performed as previously described (Hua et al., 2017; Luo et al., 2018), with minor modifications. *K. pneumoniae* strain 12092025 served as the host. Briefly, the filtrate was mixed with an equal volume of 3× LB broth and, to enrich *K. pneumoniae* phages, 1 mL of *K. pneumoniae* (OD_600_ = 0.5) was added to the mixture, followed by incubation for 8 h at 37°C with shaking. The mixture was collected by centrifugation after 8 h and passed through a 0.22-μm filter. A double-layer agar technique was used to detect and purify phages. 50 μL of filtrate and 200 μL of *K. pneumoniae* strain 12092025 were mixed with Semi-solid LB agar medium and mixtures were poured onto LB agar plates, incubating the plates overnight at 37°C. A single plaque was picked and resuspended in 500 μL of *K. pneumoniae* strain 12092025 and incubated on 200 rpm at 37°C for 2 h. Then, bacterium-phage mixture was mixed with 10 mL of the Semi-solid LB agar and poured onto LB agar plates and incubated overnight at 37°C. This process was repeated at least three times to obtain pure phage, which was named KP2025.

### Electron microscopy

A carbon copper membrane was coated with 20 μL of a pure phage sample for transmission electron microscopy (TEM). The membranes were negatively stained with 2% (*w*/*v*) phosphotungstic acid for 20 min and were then allowed to dry at room temperature. The morphology of the phage was then observed by TEM (JEM-1200EX, Japan) at an accelerating voltage of 100 kV (Mendes et al., 2014).

### Host range determination

The host range was determined using different bacterial strains kept in our laboratory as described previously (Chang et al., 2005), with the following modifications: 50 μL of a phage suspension and 200 μL of the cells from an overnight culture of bacterial were mixed with 10 mL of the Semi-solid LB agar and poured on the surface of LB agar plates, incubating the plates overnight and observe the plaques to confirm host range. 10 mL Semi-solid LB agar was mixed with 100 μL of the cells, which was then overlaid on the surface of the solidified LB agar. 10 μL (ca. 1.0 × 10^9^ PFU/mL) of a phage suspension was spotted onto the plate, which was then incubated overnight, and host range was established by bacterial lysis at the spot where the phage was deposited. Each experiment was repeated three times.

### Multiplicity of infection and one-step growth curve

The number of cells of the host bacterium *K. pneumoniae* strain 12092025 was adjusted to 1 × 10^8^ colony-forming units (CFU)/mL in a turbidimeter. According to the MOIs, the ratios between KP2025 and the host bacteria were 0.001, 0.01, 0.1, 1, 10 and 100. The phage-bacteria mixture was incubated at 37°C for 4 h and centrifuged at 8,000 rpm for 3 min. To calculate the phage titer, the supernatant was serially diluted. The MOI resulting in the release of the highest number of progeny phages was considered the ideal MOI (Wei et al., 2023).

The one-step growth curve was determined as described previously (Yuan et al., 2019; Cong et al., 2021). Briefly, *K. pneumoniae* strain 12092025 was cultured to the mid-exponential phase, collected by centrifugation at 8,000 rpm for 3 min, and resuspended in LB broth to a density of 1 × 10^8^ CFU/mL. Phage KP2025 was added at the ideal MOI of 0.01, followed by incubation at 37°C, with shaking. Measurements were taken for a total of 3.5 h. Samples were taken every 10 min for the first 40 min, then every 20 min until 120 min, and subsequently every 30 min. Phage titers were determined using the double-layer agar plate method.

### Bacteriophage DNA extraction and genome sequencing

Phenol and chloroform-isoamyl alcohol (24:1) was used to extract phage genomic DNA. A total of 200 μL of phage was 100 μL phenol, 100 μL chloroform-isoamyl alcohol mixed, vortexed for 1 min, and centrifuged at 12,000 rpm for 5 min. The above steps were repeated once. Whole-genome sequencing was performed on the Illumina NovaSeq platform.

### Whole-genome bioinformatic analysis

Raw data quality was assessed using FastQC (v.0.11.5, default settings) (Bolger et al., 2014). The sequences were assembled and spliced with SPAdes (v.3.15.3, default settings) (Bankevich et al., 2012). Open reading frames encoded in the phage genome were predicted by RAST^1^ (Aziz et al., 2008). Similarities between phage KP2025 and other phage genomes were compared using BLASTn (O'Leary et al., 2016). BLASTp^2^ (Johnson et al., 2008) was used to determine protein function and similarity. The circular map of the genome was plotted using Proksee.^3^ The gene encoding tRNA was predicted by tRNAscan-SE v.2.0^4^ (Lowe and Eddy, 1997). The online prediction platforms Virulence Finder^5^ and ResFinder^6^ (Kleinheinz et al., 2014) were employed to predict whether the genome of phage KP2025 encodes virulence and drug resistance genes. The conserved domains of LysKP213 were analyzed using the NCBI conserved domain database. Multiple sequence alignments were performed by the Clustal Omega^7^ and rendered with Espript.^8^ Three-dimensional protein models were visualized by PyMOL and WinCoot. Each online prediction tool was then run with default settings (RAST, BLASTn, BLASTp, Proksee, tRNAscan-SE, Virulence Finder, ResFinder, NCBI conserved domain database, Clustal Omega, and Espript).

### The production and purification of recombinant LysKP213 and CecA-LysKP213

The genomic DNA of phage KP2025 was used as a template for the PCR amplification of the LysKP213-encoding gene. PCR was performed using the gene-specific primer pair 5′-CGGGATCCATGG CTAGAGTAGTAGAT-3′ (forward) and 5′-CCCAAGCTTGTTGTA AGCATCAAGT-3′ (reverse). *Bam*H I and *Hin*d III restriction sites, present, respectively, at the 5′ ends of the forward and reverse primers (underlined), were used for cloning the amplified sequences into the pET-28a vector. The PCR conditions were as follows: pre-denaturation at 94°C for 5 min, followed by 30 cycles of denaturation at 94°C for 30 s, annealing at 55°C for 45 s, and extension at 72°C for 90 s, with a final extension step at 72°C for 10 min, 30 cycles in total. The PCR products were detected using 1% agarose gel electrophoresis and the LysKP213-encoding gene size was found to contain 501 bp. *Escherichia coli* BL21 (DE3) carrying pET-28a-LysKP213 was used for the expression of recombinant LysKP213 (Wang et al., 2022). CecA-LysKP213 was constructed by fusing CecA (NCBI PRF 0708214A) to the 5′ end of LysKP213 with the (GGGGS)_3_ linker (Hong et al., 2022). Specifically, cells expressing the recombinant protein were cultured in LB broth containing 50 μg/mL kanamycin at 37°C with shaking. At an OD_600_ of 0.5, isopropyl-β-D-thiogalactopyranoside (IPTG) was added at the final concentration of 0.5 mM, and the bacteria were incubated at 28°C (LysKP213) or 22°C (CecA-LysKP213) for 8 h with shaking. Following centrifugation at 12,000 rpm for 10 min, the bacterial pellet was resuspended in 10 mL of 5 mM imidazole containing NaH_2_PO_4_ and NaCl supplemented with 10 μL of protease inhibitor. Ultrasonication was performed with an ultrasonic crusher (135 W, 70% power, 3 s on and 3 s off, for a total of 30 min). Recombinant LysKP213 and CecA-LysKP213 were Ni-affinity purified using a His Trap HP column. The proteins were collected by eluting with a gradient of 5–250 mM imidazole and dialyzed against 20 mM Tris–HCl and 10% glycerol (pH 7.4). Recombinant LysKP213 and CecA-LysKP213 were validated using 12% SDS–PAGE.

### Analysis of the antibacterial activity of LysKP213 and CecA-LysKP213

As described previously, the antibacterial activity of LysKP213 was performed by Lu et al. (2021). Briefly, the bacteria were cultured at 37°C with shaking to an OD_600_ of 0.6–0.8 and centrifuged at 12,000 rpm for 1 min. The bacterial pellet was washed once with 20 mM Tris–HCl and treated with 0.5 M EDTA for 5 min, washed again with Tris–HCl, centrifuged at 12,000 rpm for 1 min, and finally resuspended in Tris–HCl. After the addition of LysKP213, the bacteria were incubated at 37°C. The standard turbidity measurement method was used and the OD_600_ was recorded using a plate reader. Additionally, serially diluted bacteria were spotted on LB agar, and the number of CFUs was calculated. The bacteria inhibitory activity of LysKP213 was calculated as [OD_600_ (bacteria only) − OD_600_ sample (endolysin added)]/[OD_600_ (bacteria only)] (Plotka et al., 2015).

The antibacterial activity of CecA-LysKP213 was determined using *K. pneumoniae* strain 12092025, *P. aeruginosa* strain PAO1, *A. baumannii* strain 12091082, and *E. coli* strain B5 as described previously (Lim et al., 2022). Briefly, after culturing to the exponential phase (OD_600_ = 0.6–0.8), the bacteria were collected by centrifugation at 12,000 rpm for 1 min and adjusted to 1 × 10^8^ CFU/mL in 20 mM Tris–HCl after washing. The bacteria were then mixed with CecA-LysKP213 (21 μg/mL), incubated at 37°C for 2 h, serially diluted, and spotted on LB agar to calculate the number of bacteria.

### Analysis of the effects of pH, NaCl concentration, human serum, and temperature on LysKP213 activity

To study the effect of temperature on endolysin activity, the endolysin was preincubated at various temperatures (4–100°C) for 30 min. *K. pneumoniae* strain 12092025 was washed once with 20 mM Tris–HCl (pH 7.4) and treated with 0.5 M EDTA for 5 min, washed again with Tris–HCl, centrifuged at 12,000 rpm for 1 min, and finally resuspended in Tris–HCl. All groups were then added to the cell preparations for 30 min at 37°C. After incubation, the OD_600_ value was determined (Yu et al., 2019).

To investigate the effect of pH on the activity of LysKP213, 0.5 M EDTA-treated *K. pneumoniae* strain 12092025 was resuspended in buffer solutions of different pH (20 mM citrate buffer pH 4–5, 20 mM imidazole-HCl buffer pH 6, and 20 mM tris–HCl buffer pH 7–12) and incubated with the endolysin for 30 min at 37°C. After incubation, the OD_600_ value was determined (Yu et al., 2019).

The activity of LysKP213 at NaCl concentrations from 0 to 500 mM was measured at optimal pH conditions (Kim et al., 2020). Some modifications were made to the endolysin antibacterial activity method in different human serum described by Chen et al. (2021).

To test the endolysin antibacterial activity in human serum, *K. pneumoniae* strain 12092025 was washed once with 20 mM Tris–HCl (pH 7.4) and treated with 0.5 M EDTA for 5 min, washed again with Tris–HCl, centrifuged at 12,000 rpm for 1 min, and finally resuspended in Tris–HCl. Then, cells were treated with 21 μg/mL LysKP213 in the presence of 1–10% human serum (Sigma-Aldrich, Shanghai, China) at 37°C for 30 min. Endolysin treatment in the absence of human serum served as the control. After incubation, the OD_600_ value was determined. The antibacterial activity of LysKP213 was calculated as [OD_600_ (bacteria only) − OD_600_ sample (endolysin added)]/[OD_600_ (bacteria only)]. Each experiment was repeated three times (Chen et al., 2021).

### Assessment of thermal stability

Some modifications were made to the endolysin thermal stability method described by Plotka et al. (2014). Briefly, aliquots of endolysin LysKP213and hen egg white lysozyme (HEWL; 21 μg/mL) were heated at 95°C for increasing durations in a Super Gradient Thermal Cycler (LongGene) or autoclaved at 121°C for 30 min. Then, 0.5 M EDTA-treated *K. pneumoniae* strain 12092025 incubated with the endolysin for 30 min at 37°C. After incubation, the OD_600_ value was determined.

### Determination of the minimum inhibitory concentration (MIC)

The CLSI-recommended broth microdilution method was used to determine the MIC (the lowest concentration at which an antibiotic inhibits bacterial growth) of the antibiotic polymyxin B in Mueller Hinton broth (MHB). The obtained MIC value was used for the subsequent combination experiment.

### The combination of LysKP213 with Polymyxin B

Some modifications were made to the endolysin LysKP213 combined with polymyxin B method described by Sitthisak et al. (2023). *Klebsiella pneumoniae* strain 12092025, *A. baumannii* strain 12091082, *P. aeruginosa* strain PAO1, and *E. coli* strain B5 were used as bacterial substrates. The bacteria were cultured in LB broth and the turbidity of the bacterial suspension was measured using a densitometer. The turbidity was corrected to an equivalent 0.5 McFarland standard or approximately 1 × 10^8^ CFU/mL, and the cell suspension was diluted 50 times with 2× MHB. Aliquots of 100 μL were transferred to 96-well plates, and 50 μL of LysKP213 at different concentrations (0–85 μg/mL) and 50 μL of different concentrations of polymyxin B were added to the wells. The bacteria were then cultured in an incubator at 37°C protected from light for 16 h, following which the OD_600_ was determined using a spectrophotometer. Each experiment was repeated three times.

### *Galleria mellonella* larval infection model

The *Galleria mellonella* larval model study was conducted following the procedures by Kim et al. with some minor modifications (Kim et al., 2018) and referring to other *Galleria mellonella* larval studies (Chen et al., 2021). Briefly, *Galleria mellonella* was used as the experimental animal. *G. mellonella* larvae with a length of 20–25 mm and weighing 250–350 mg are stored in a dark environment at 4°C for 24 h before infection. The larvae were injected through the last right proleg with 10 μL of a bacterial solution containing 1 × 10^7^ CFU/mL of *K. pneumoniae* strain 12092025 or 1 × 10^8^ CFU/mL of *A. baumannii* strain 12091082, *P. aeruginosa* strain PAO1, or *E. coli* strain B5. These doses were previously determined to yield 80% mortality after 96 h. The infected larvae were divided into the following six groups (10 larvae per group): (i) a control group, which received phosphate-buffered saline (PBS); (ii) a polymyxin B treatment group, which received either 14 μg/mL polymyxin B (for *K. pneumoniae* strain 12092025-infected larvae), 12 μg/mL polymyxin B (for *P. aeruginosa* strain PAO1-infected larvae), 10 μg/mL polymyxin B (for *A. baumannii* strain 12091082-infected larvae), or 8 μg/mL polymyxin B (for *E. coli* strain B5-infected larvae); (iii) a LysKP213 treatment group, which received 21 μg/mL LysKP213; (iv) a combination treatment group, which received polymyxin B plus LysKP213 treatment; (v) a CecA (GL Biochem (Shanghai) Ltd., China) treatment group, which received 3 μg/mL CecA; and (vi) a CecA-LysKP213 treatment group, which received 21 μg/mL CecA-LysKP213. All treatments were administered via injection (10 μL) into the last left proleg 30 min following infection. After treatment, the larvae of each group were placed in Petri dishes and cultured in darkness at 37°C for 96 h. Mortality was assessed every 12 h. All the experiments were independently performed three times.

### Statistical analysis

GraphPad Prism v.8.0 was used for data analysis. Differences between datasets were evaluated using two-tailed Student’s *t*-tests; for survival experiments, a log-rank (Mantel-Cox) test was employed. Data are presented as means ± SD, with *p*-values <0.05 being considered significant.

## Results

### Biological characteristics and host range of phage KP2025

**Figure 1:**
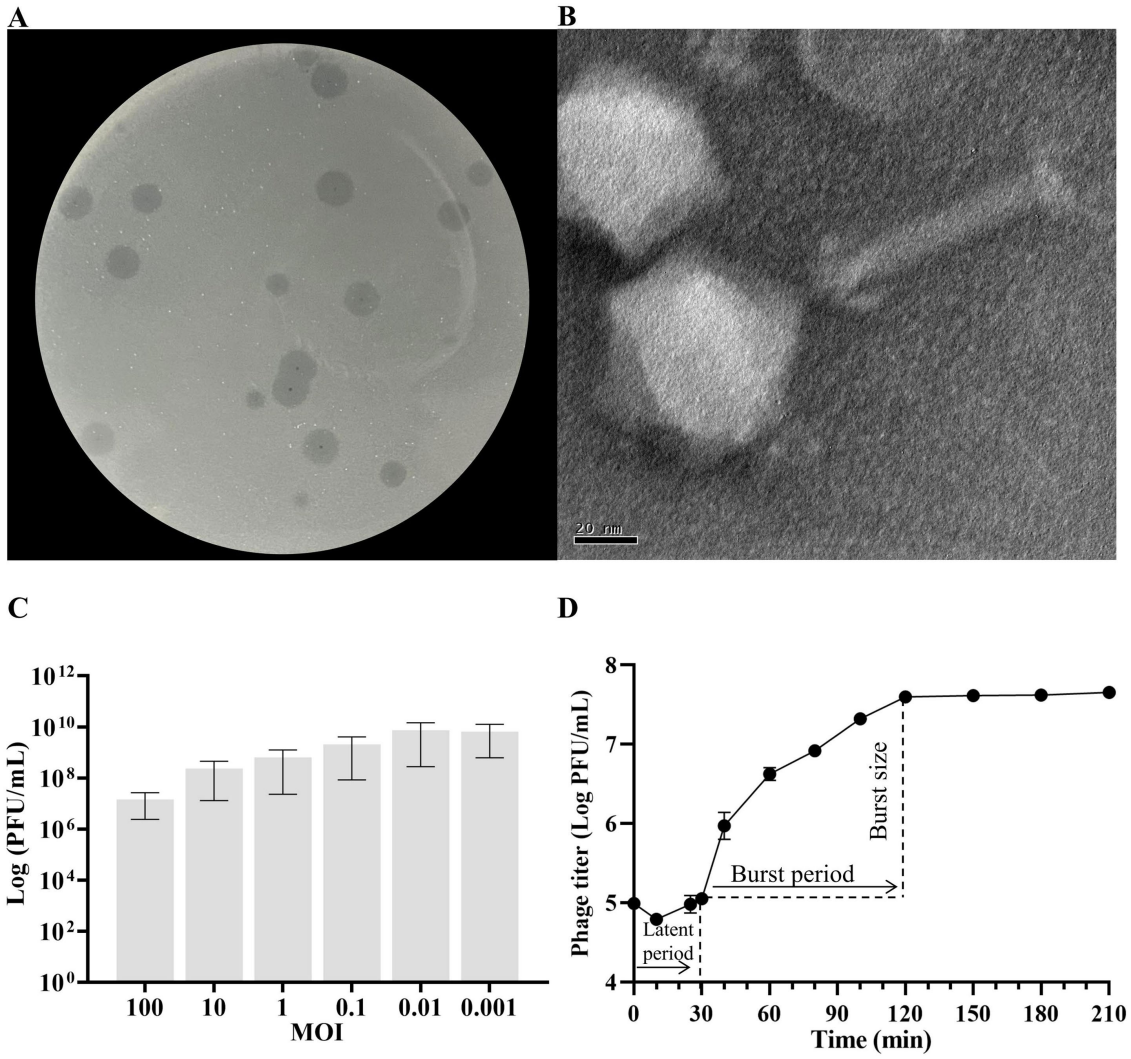
Biological characterization of *Klebsiella pneumoniae* phage KP2025. **(A)** Clear plaques of phage KP2025 on double-layer agar. **(B)** Transmission electron micrograph of phage KP2025. Scale bar = 20 nm. **(C)** Titers of phage KP2025 produced at different multiplicities of infection (MOIs). Values represent means ± SD (*n* = 3). **(D)** One-step growth curve for phage KP2025. Values represent means ± SD (*n* = 3).

The isolated phage KP2025 was initially screened against *K. pneumoniae* strain 12092025 through a spot test. KP2025 produced clear plaques of similar morphology (Figure 1A). Transmission electron micrograph analysis showed that phage KP2025 has an icosahedral head and slender tail, characteristic of *Myoviridae* (Figure 1B).

The MOI that yielded the highest phage titer among the assessed MOIs was 0.01 (Figure 1C). A one-step growth curve reflects the important parameters of phage growth, including the latent period, burst size, and release period. As illustrated in Figure 1D, the latent and rising periods of KP2025 lasted for 30 and 90 min, respectively, and the average burst size (the number of phage particles released by each infected host cell) was 100 ± 10 PFU/mL. KP2025 exhibited good lytic properties against the clinical *K. pneumoniae* and *E. coli* strains, as evidenced by the results of the host range assay (Supplementary Table S1).

### Identification of LysKP213 from the genome of phage KP2025

**Figure 2:**
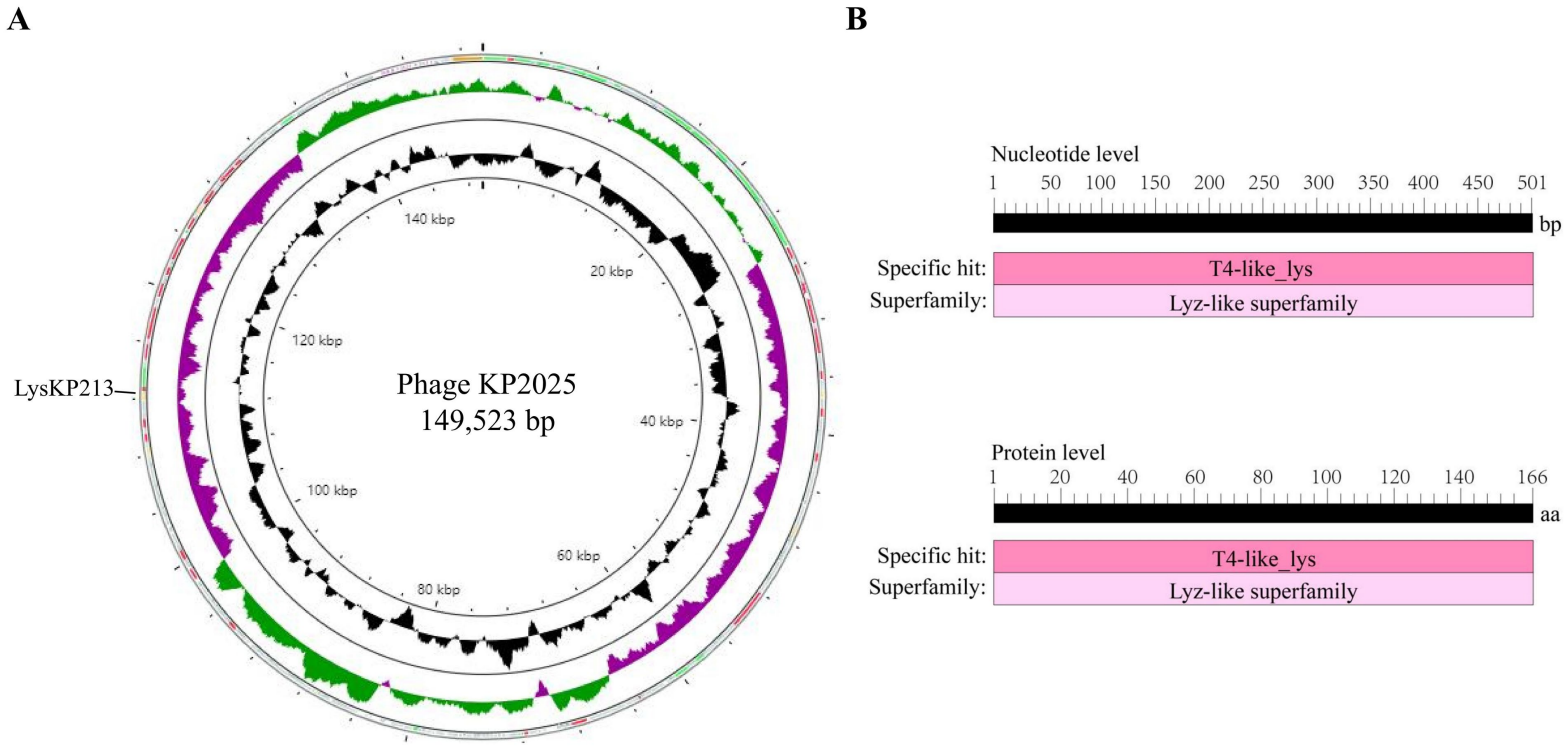
Whole-genome sequencing of phage KP2025 and identification of the endolysin LysKP213. **(A)** The genomic map of phage KP2025. The inner to outer circles were as: genome size (with an interval of 4 kb); GC content; GC skew [(G − C)/(G + C)]; and open reading frames (ORFs). **(B)** Conserved domain analysis of LysKP213 at the nucleic acid and protein levels.

The genome of phage KP2025 contains 149,523 bp and has a GC content of 40.4% (GenBank accession no. PP919962). The genome contains 275 predicted open reading frames (ORFs) and 19 tRNAs (Supplementary Table S2). BLASTp analysis revealed that ORF213 (length: 501 bp) encodes a putative endolysin, which we named LysKP213 (Figure 2A). This endolysin contains a conserved T4-like_lys domain (NCBI domain architecture ID: 10091399; conserved domain accession: cd00735) and belongs to the Lyz-like superfamily (Figure 2B).

**Figure 3:**
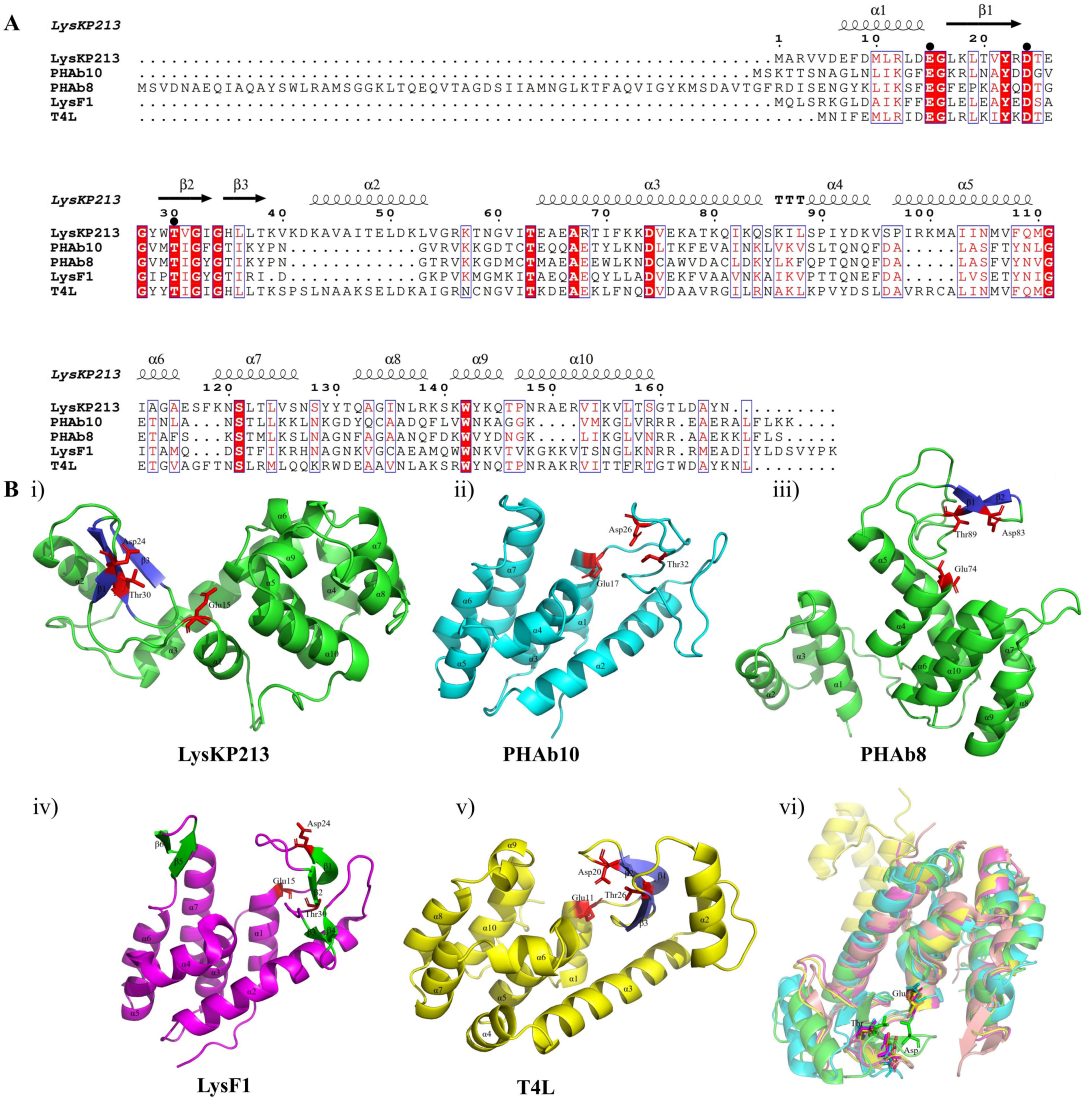
Multiple sequence alignment and structure analysis of the endolysin LysKP213. **(A)** Multiple sequence comparison between LysKP213 and four representative members of the Lyz-like superfamily; black circles indicate key catalytic sites of the enzyme. **(B)** Three-dimensional structure of LysKP213 **(i)**, PHAb10 **(ii)**, PHAb8 **(iii)**, LysF1 **(iv)**, and T4L **(v)**. **(vi)** Structure superposition of LysKP213 and four classical T4L-like lysozymes. LysKP213 is shown in cyan, PHAb10 in magenta, PHAb8 in yellow, T4L in green, and LysF1 in pink. Residues in the conservative catalytic triad are shown as sticks.

In addition, we performed a multiple sequence alignment between LysKP213 and four representative members of the Lyz-like superfamily, including the peptidoglycan hydrolases from the *A. baumannii* 10 (PHAb10), the peptidoglycan hydrolases from the *A. baumannii* 8 (PHAb8), the *E. coli* O157:H7 phage FAHEc1 endolysin (LysF1), and the well-studied T4 lysozyme (T4L) (Weaver and Matthews, 1987; Love et al., 2021; Zhang et al., 2024). The result showed that they possessed the conserved Glu-Asp-Thr catalytically active sites and are mainly composed of α-helices and β-folds, characteristic of the Lyz-like superfamily (Figure 3A; Supplementary Table S3). Further three-dimensional structure analysis indicated that LysKP213, PHAb10, PHAb8, and LysF1 superimposed well with T4L, especially in the conserved Glu-Asp-Thr catalytically active sites (Figure 3B). Thus, these results indicated that LysKP213, PHAb10, PHAb8, and LysF1 were highly similar to T4L and might share similar catalytic mechanisms of action.

### Molecular cloning, expression, purification, and activity validation of recombinant LysKP213

**Figure 4:**
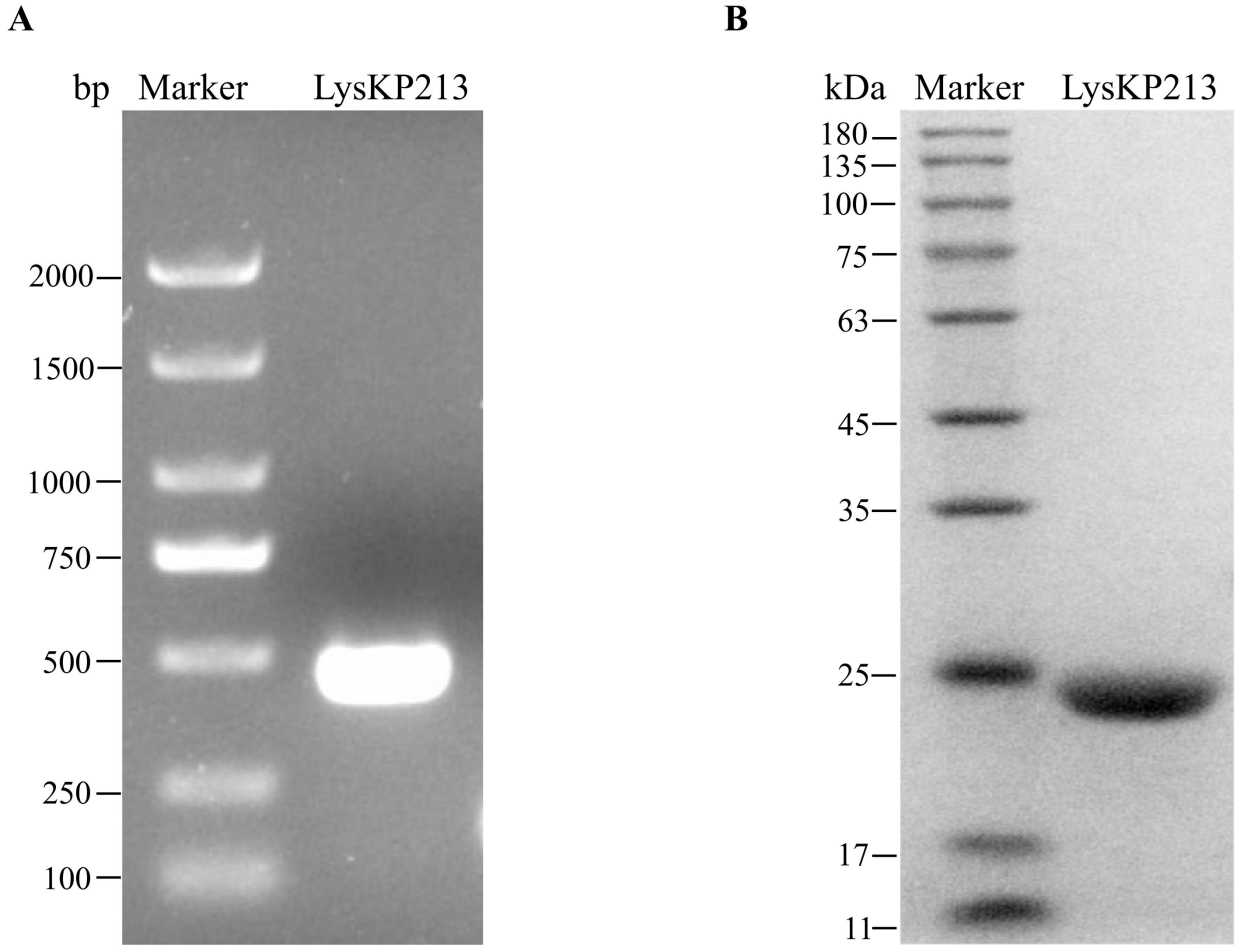
Gene cloning, expression, and purification of the endolysin LysKP213. **(A)** The genome of bacteriophage KP2025 was used as a template for the PCR amplification of the LysKP213-encoding gene. **(B)** IPTG (final concentration 0.5 mM) was used to induce the overexpression of recombinant LysKP213; the final purified endolysin LysKP213 was analyzed using 12% SDS–PAGE.

The LysKP213-encoding gene segment was amplified from the genome of phage KP2025 by PCR (Figure 4A). The IPTG-induced expression of recombinant endolysin LysKP213 was carried out in genetically engineered *E. coli* BL21 (DE3). Subsequently, a HisTrap affinity column was used to obtain purified LysKP213. The band size was further confirmed by 12% SDS–PAGE (Figure 4B).

**Figure 5:**
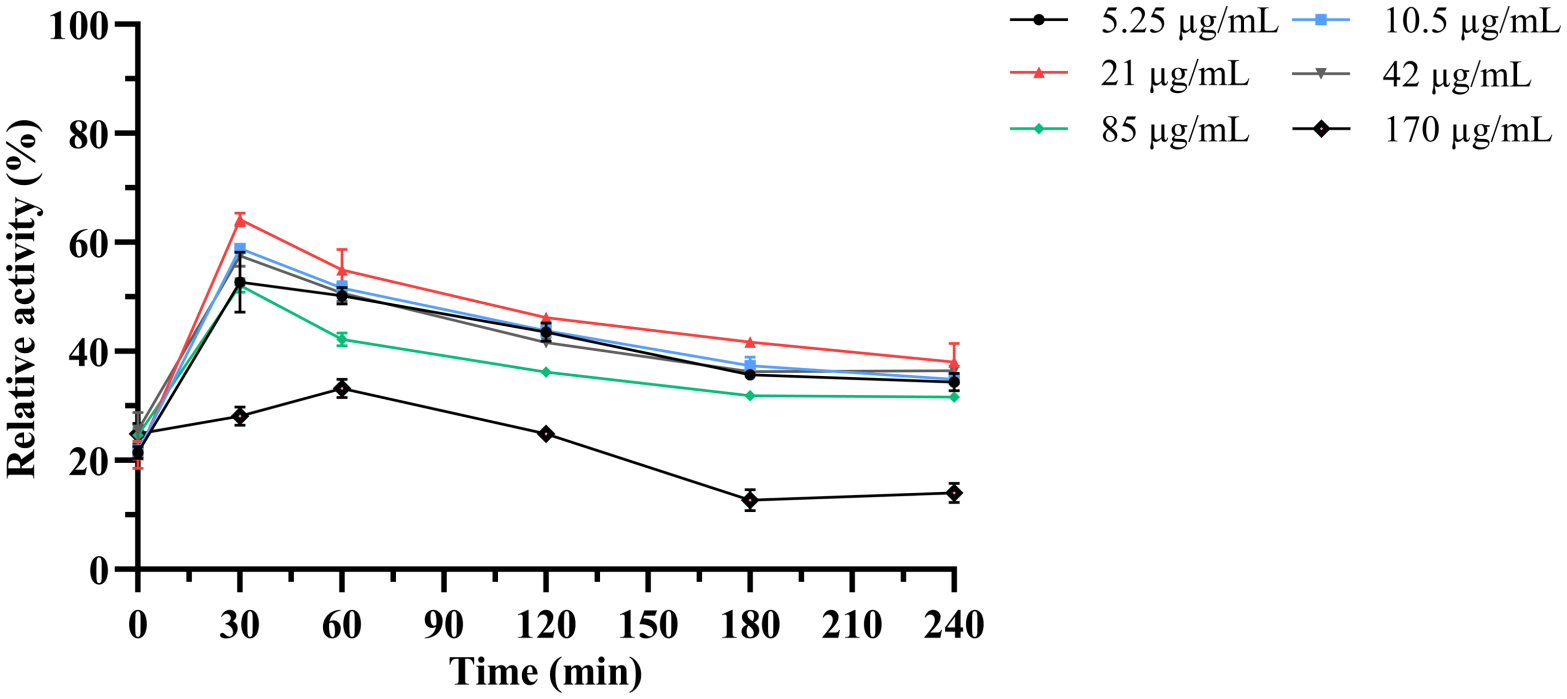
The antimicrobial activity of different concentrations of endolysin LysKP213 against *K. pneumoniae* strain 12092025. Each test were repeated three times. The values are the means and standard deviations from triplicate assays.

Next, the antimicrobial activity of the purified LysKP213 was assessed using the turbidity reduction assay. *K. pneumoniae* strain 12092025 served as the host strain and phage KP2025 treated with 0.5 M EDTA served as the test strain. The results showed that the antimicrobial activity of LysKP213 increased in a concentration-dependent manner; however, this activity decreased at concentrations over 21 μg/mL. In addition, the highest antimicrobial activity of LysKP213 was observed at the concentration of 21 μg/mL after 30 min of incubation (Figure 5).

### The influence of temperature, pH, human serum, and NaCl on the lytic activity of LysKP213

**Figure 6:**
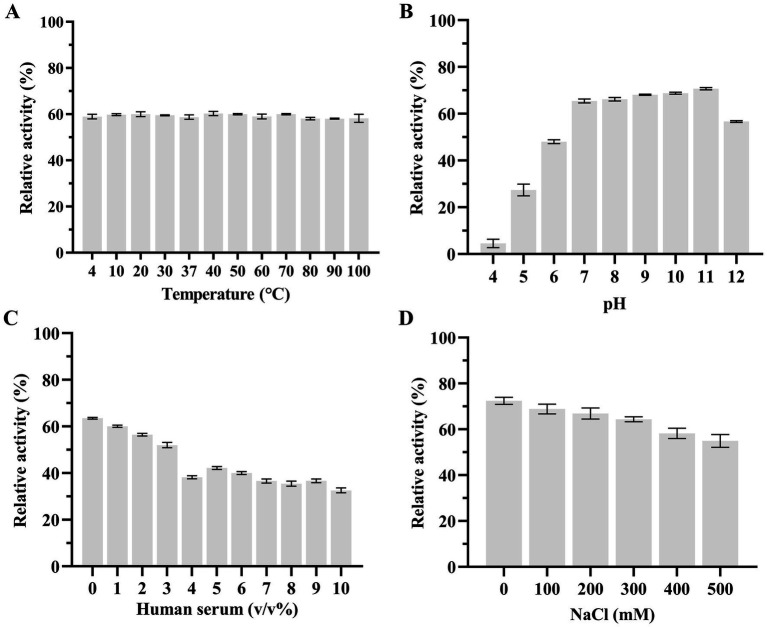
The effects of temperature **(A)**, pH **(B)**, human serum **(C)**, and NaCl **(D)** on the lytic activity of the endolysin LysKP213. *Klebsiella pneumoniae* strain 12092025, the host strain for bacteriophage KP2025, was used as an effector substrate. In addition, the maximum activity of LysKP213 was set as 100% under various test conditions to facilitate comparisons. Each experiment was performed in triplicate. The error bars represent the SD.

Subsequently, we used the host strain of bacteriophage KP2025, *K. pneumoniae* strain 12092025, as the effector substrate to measure the effects of temperature, pH, human serum, and salinity (NaCl concentrations) on LysKP213 activity. We found that the thermostability of LysKP213 was very broad, ranging from 4 to 100°C (Figure 6A). The pH values tested ranged from 4 to 12 and LysKP213 had relatively high lytic activity in the 7–11 pH range (Figure 6B). The concentration of human serum significantly affected the activity of LysKP213; nevertheless, relatively high lytic activity was observed at concentrations between 1 and 3% (Figure 6C). The most suitable pH identified in the pH test was used to assess the effect of salinity (NaCl concentration) on the activity of LysKP213. We noted that LysKP213 retained 60% relative activity against *K. pneumoniae* strain 12092025 at NaCl concentrations of up to 500 mM (Figure 6D).

**Figure 7:**
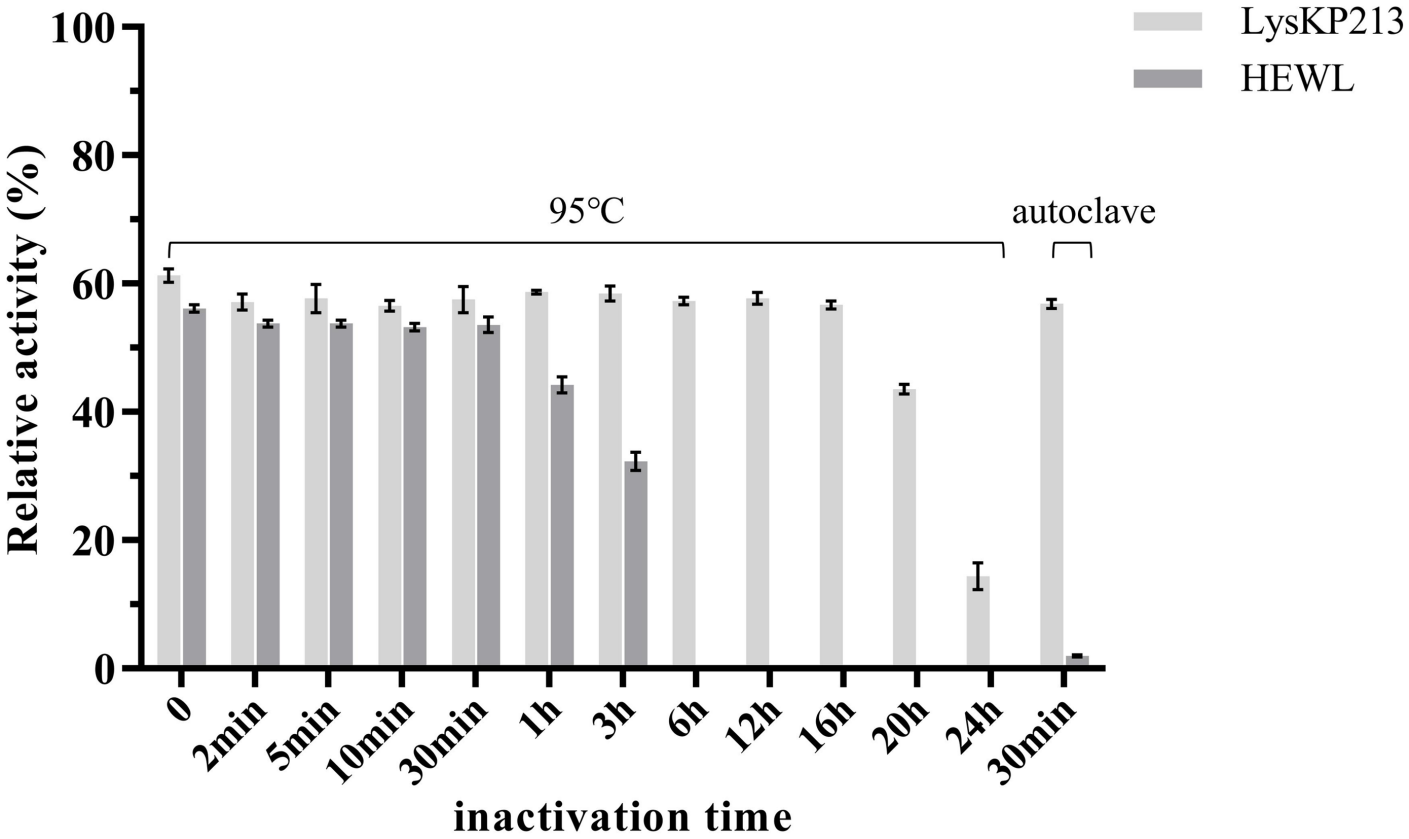
Examination of the thermal stability of the endolysin LysKP213. LysKP213 (21 μg/mL) and hen egg white lysozyme (HEWL) (21 μg/mL) were incubated at 95°C for 0–24 h or autoclaved for 30 min at 121°C, following which their activity against *Klebsiella pneumoniae* strain 12092025 cells was measured. The activities of LysKP213 and HEWL are indicated as percentages relative to untreated samples (0 min). Each experiment was performed in triplicate. The error bars indicate the standard deviation.

We further estimated the thermostability of endolysin LysKP213. For this, LysKP213 was incubated for increasing durations (up to 24 h) at 95°C or autoclaved at 121°C for 30 min, followed by an activity assay (Figure 7). The results indicated that LysKP213 is highly thermostable. After incubation at 95°C for 20 h, LysKP213 retained 44.4% of its lytic activity. Furthermore, LysKP213 retained 15.8% residual activity after 24 h at 95°C, and 57.5% residual activity after 30 min at 121°C. Meanwhile, HEWL, used as a control, was completely inactivated after 6 h at 95°C.

### Analysis of the lytic activity spectrum of LysKP213

**Figure 8:**
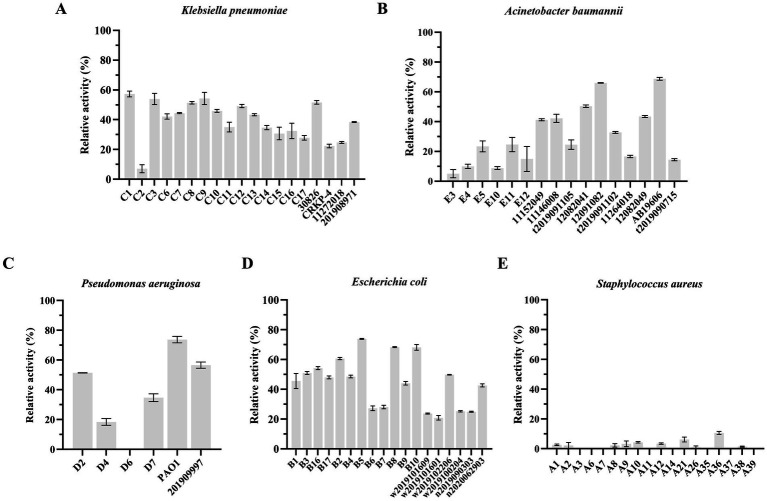
The lytic activity spectrum of endolysin LysKP213 against *Klebsiella pneumoniae* **(A)**, *Acinetobacter baumannii* **(B)**, *Pseudomonas aeruginosa* **(C)**, *Escherichia coli* **(D)**, and *Staphylococcus aureus* **(E)** as determined using the standard turbidity measurement method. Each experiment was performed in triplicate.

Like other phage-encoded endolysins, LysKP213 could not directly lyse Gram-negative bacteria owing to the protection afforded by the OM. Thus, *K. pneumoniae*, *A. baumannii*, *P. aeruginosa*, and *E. coli* were treated with EDTA to remove the OM before the addition of LysKP213. We observed that LysKP213 could lyse most of the Gram-negative bacterial strains tested as determined by the measurement of turbidity (Figure 8; Supplementary Table S4). Moreover, LysKP213 exhibited substantial lytic activity against *K. pneumoniae* strain 12092025, *A. baumannii* strain 19606, *P. aeruginosa* strain PAO1, and *E. coli* strain B5; however, LysKP213 had no lytic activity against the Gram-positive bacterium *Staphylococcus aureus* (Figure 8; Supplementary Table S4).

**Figure 9:**
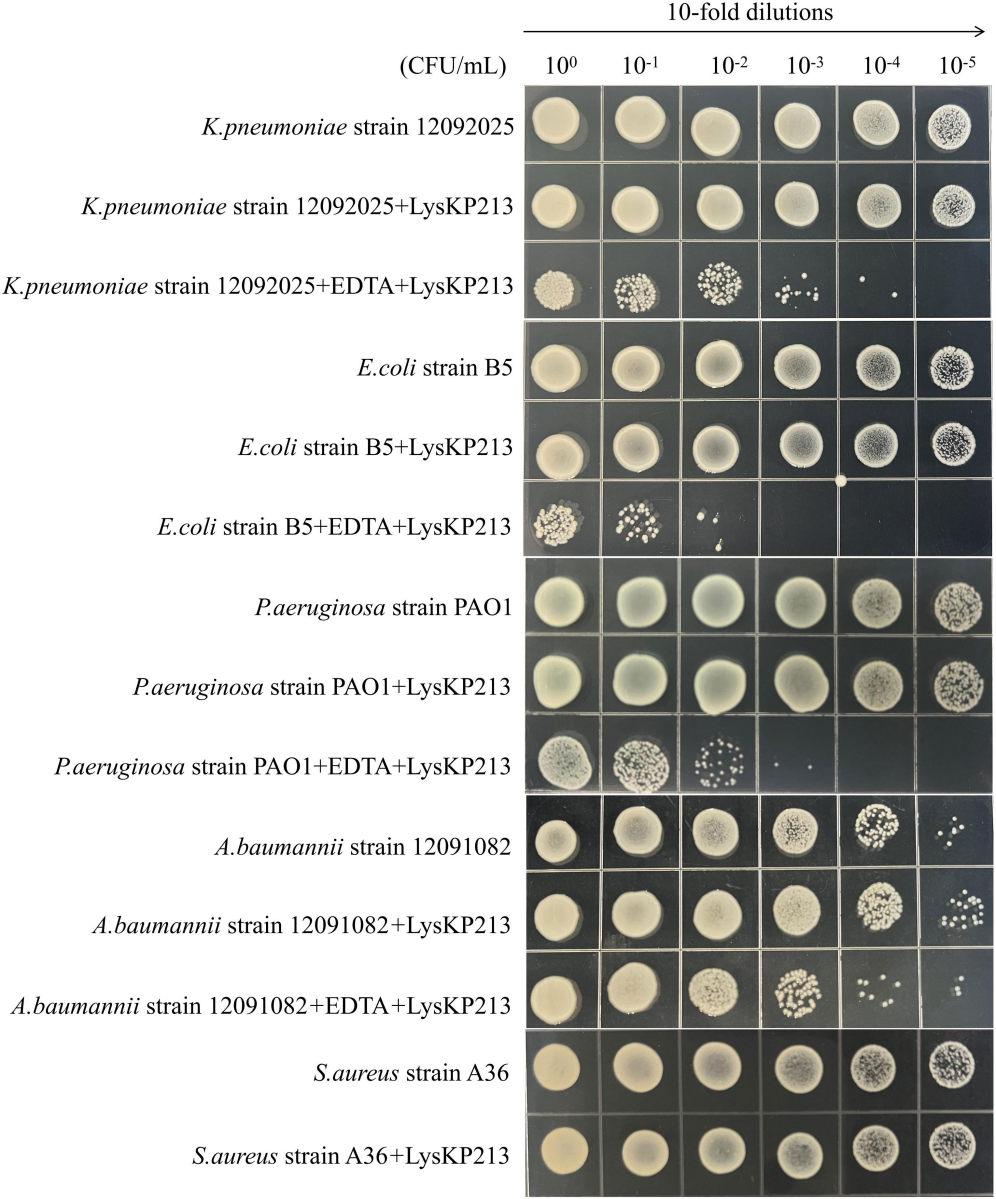
Representative images depicting bacterial cell viability before and after LysKP213 treatment. To measure viability, 10-fold dilutions of log-phase bacterial cells (*Klebsiella pneumoniae* strain 12092025, *Pseudomonas aeruginosa* strain PAO1, *Acinetobacter baumannii* strain 12091082, and *Escherichia coli* strain B5) that were untreated, treated with EDTA, or treated with EDTA plus LysKP213 were spotted onto agar plates. Additionally, 10-fold dilutions of log-phase *Staphylococcus aureus* strain A36 cells, untreated or treated with LysKP213, were also spotted on agar plates. LysKP213 in combination with EDTA reduced the numbers of viable *K. pneumoniae*, *P. aeruginosa*, *A. baumannii*, and *E. coli* cells by 1–3 log units; however, LysKP213 treatment did not affect *S. aureus* strain A36. The numbers above the colony represent the dilution ratio.

We further investigated the lytic activity of LysKP213 against *K. pneumoniae* strain 12092025, *P. aeruginosa* strain PAO1, *A. baumannii* strain 12091082, *E. coli* strain B5, and *S. aureus* strain A36 using CFU reduction analysis. Compared to the control group, the Gram-negative bacterial cells showed a significant reduction in the number of CFUs after treatment with LysKP213 (Figure 9). These findings were consistent with those observed using the turbidity measurement method described above and indicated that, when the OM is removed, LysKP213 has relatively broad lytic activity against most Gram-negative bacteria, including *K. pneumoniae*, *A. baumannii*, *P. aeruginosa*, and *E. coli*.

### The synergistic effects of endolysin LysKP213 in combination with polymyxin B *in vitro*

**Figure 10:**
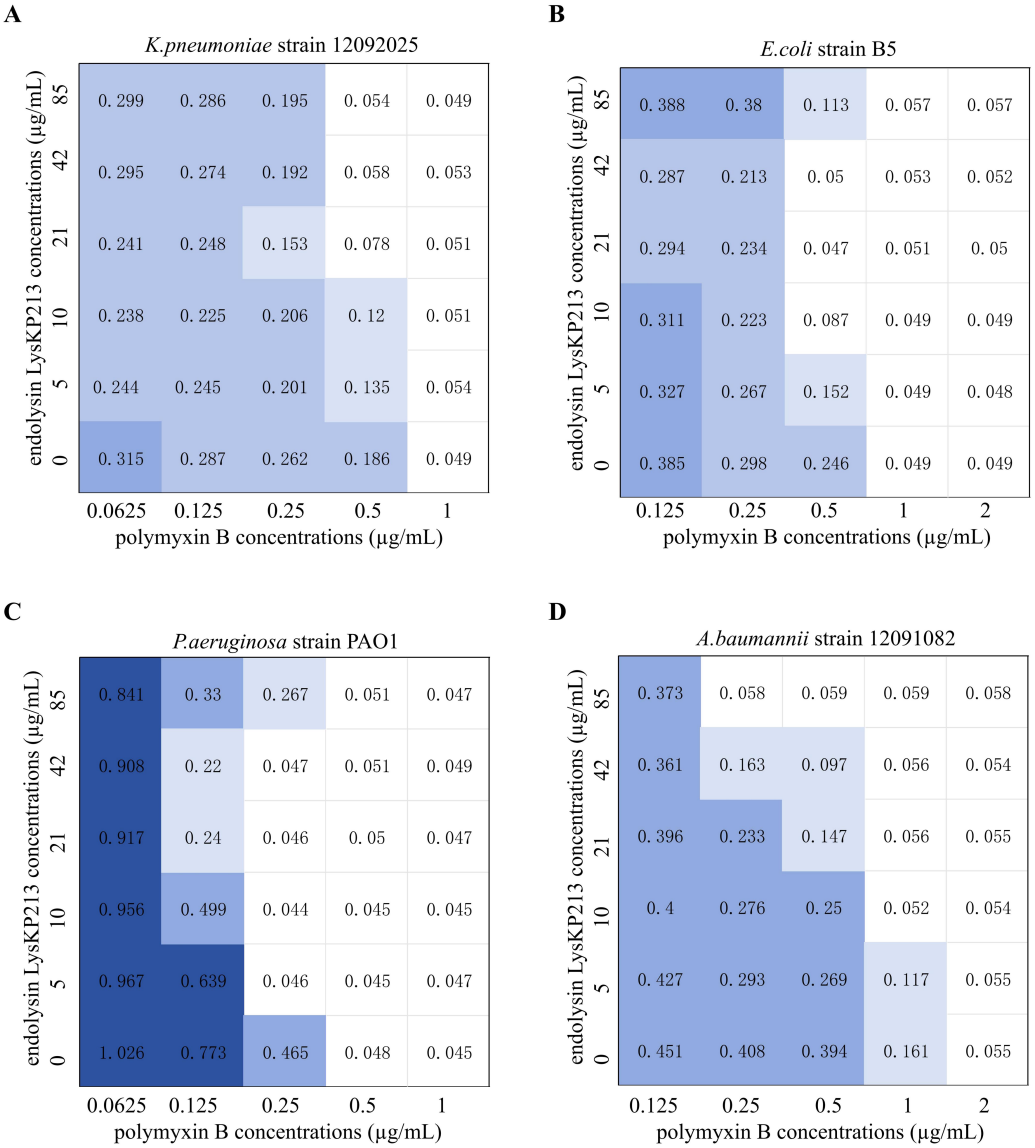
The microdilution checkboard assay for the synergistic effects of LysKP213 in combination with polymyxin B on *Klebsiella pneumoniae* strain 12092025 **(A)**, *Escherichia coli* strain B5 **(B)**, *Pseudomonas aeruginosa* strain PAO1 **(C)**, and *Acinetobacter baumannii* strain 12091082 **(D)**. The results are representative of at least three independent experiments.

The antibiotic colistin disrupts the OM of Gram-negative bacteria. Recently, it was shown that colistin has synergistic effects against Gram-negative bacteria when administered in combination with phage lysins (Thummeepak et al., 2016). Accordingly, we next evaluated the putative synergistic effect of the endolysin LysKP213 in combination with polymyxin B *in vitro* using the microdilution checkboard assay. First, we measured the MICs of polymyxin B alone against *K. pneumoniae* strain 12092025, *E. coli* strain B5, *P. aeruginosa* strain PAO1, and *A. baumannii* strain 12091082 (Supplementary Table S5). Next, we measured the effects of LysKP213 in combination with polymyxin B and found that this combination inhibited the viability of the four bacterial strains at the concentrations of 0.5 and 21 μg/mL, 0.5 and 10 μg/mL, 0.25 and 5 μg/mL, and 0.25 and 85 μg/mL, respectively (Figure 10). The addition of LysKP213 yielded a two-fold reduction in the MICs of polymyxin B against *K. pneumoniae* strain 12092025, *E. coli* strain B5, and *P. aeruginosa* strain PAO1, and an eight-fold reduction in the polymyxin B MIC against *A. baumannii* strain 12091082 (Figure 10).

### The antibacterial activity of endolysin LysKP213 in combination with polymyxin B *in vivo*

**Figure 11:**
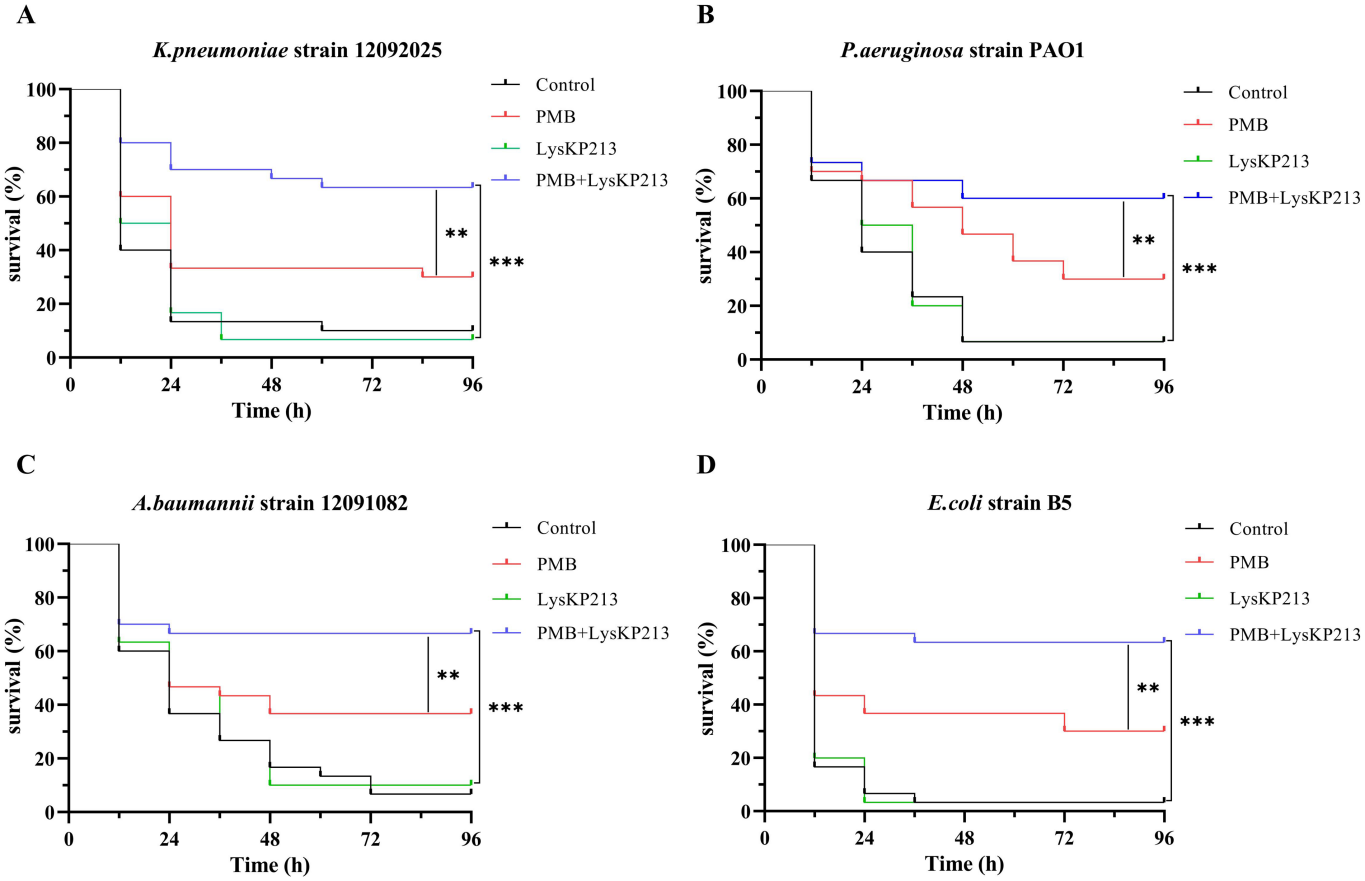
The antibacterial activity of endolysin LysKP213 in combination with polymyxin B *in vivo*. Survival curves for *Galleria mellonella* larvae infected with *Klebsiella pneumoniae* strain 12092025 **(A)**, *Pseudomonas aeruginosa* strain PAO1 **(B)**, *Acinetobacter baumannii* strain 12091082 **(C)**, and *Escherichia coli* strain B5 **(D)**. *G. mellonella* larvae were separately infected with each of the four bacterial strains and then treated with PBS (control), polymyxin B (14 μg/mL), LysKP213 (21 μg/mL), or polymyxin B (14 μg/mL) in combination with LysKP213 (21 μg/mL). **p < 0.01, ***p < 0.001.

Next, we evaluated the antibacterial activity of the endolysin LysKP213 in combination with polymyxin B *in vivo* using a *G. mellonella* larvae infection model with *K. pneumoniae* strain 12092025, *P. aeruginosa* strain PAO1, *A. baumannii* strain 12091082, and *E. coli* strain B5. The survival rate of *G. mellonella* larvae infected with any of the four tested strains was significantly higher after combination treatment than after treatment with polymyxin B or LysKP213 alone (**p < 0.01 and ***p < 0.001, respectively) (Figure 11).

### The synergistic effects of the engineered fusion protein CecA-LysKP213 *in vitro*

**Figure 12:**
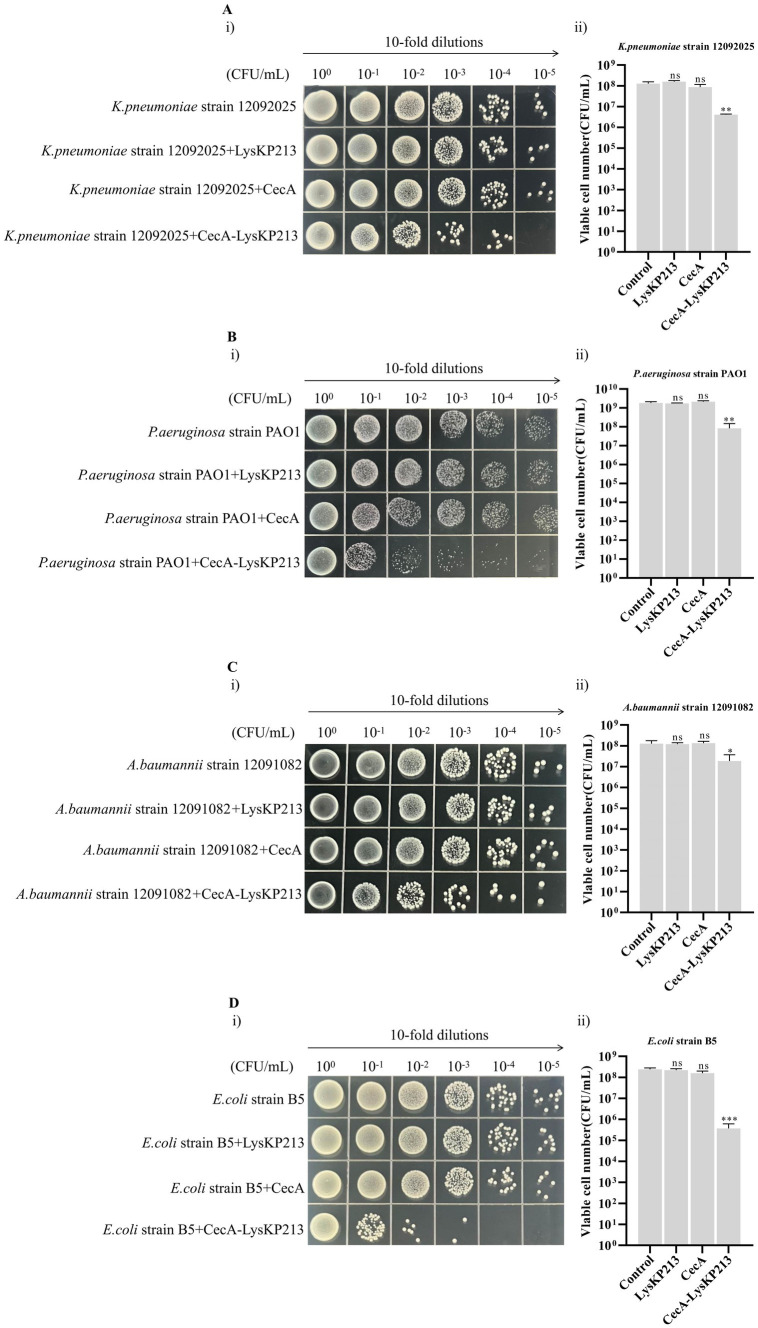
The antibacterial activity of CecA-LysKP213 against Gram-negative bacteria *in vitro*. The antibacterial activity of CecA-LysKP213 was tested against *Klebsiella pneumoniae* strain 12092025 **(A)**, *Pseudomonas aeruginosa* strain PAO1 **(B)**, *Acinetobacter baumannii* strain 12091082 **(C)**, and *Escherichia coli* strain B5 **(D)** using a colony forming unit (CFU) reduction assay. The bacteria were treated with PBS (control), CecA-LysKP213 (21 μg/mL), CecA (3 μg/mL), or LysKP213 (21 μg/mL). The experiments were repeated three times and the data are presented as means ± SD. *p < 0.05, **p < 0.01, ***p < 0.001; n.s., not significant.

Phage endolysin has difficulty reaching the peptidoglycan layer of Gram-negative bacteria owing to the presence of an OM. Here, we tested whether the inclusion of the membrane-destabilizing peptide, CecA, would increase membrane permeability and thus enhance the antibacterial activity of LysKP213. For this, we fused CecA to the N-terminus of LysKP213 (CecA-LysKP213) (Supplementary Figure S1) and found that CecA-LysKP213 reduced the numbers of *K. pneumoniae* strain12092025, *P. aeruginosa* strain PAO1, *A. baumannii* strain 12091082, and *E. coli* strain B5 cells by 1–2 log units (Figure 12). This demonstrated the antibacterial activity of LysKP213 against Gram-negative bacteria *in vitro* was improved when it was fused to CecA.

### The antibacterial activity of the engineered fusion protein CecA-LysKP213 *in vivo*

**Figure 13:**
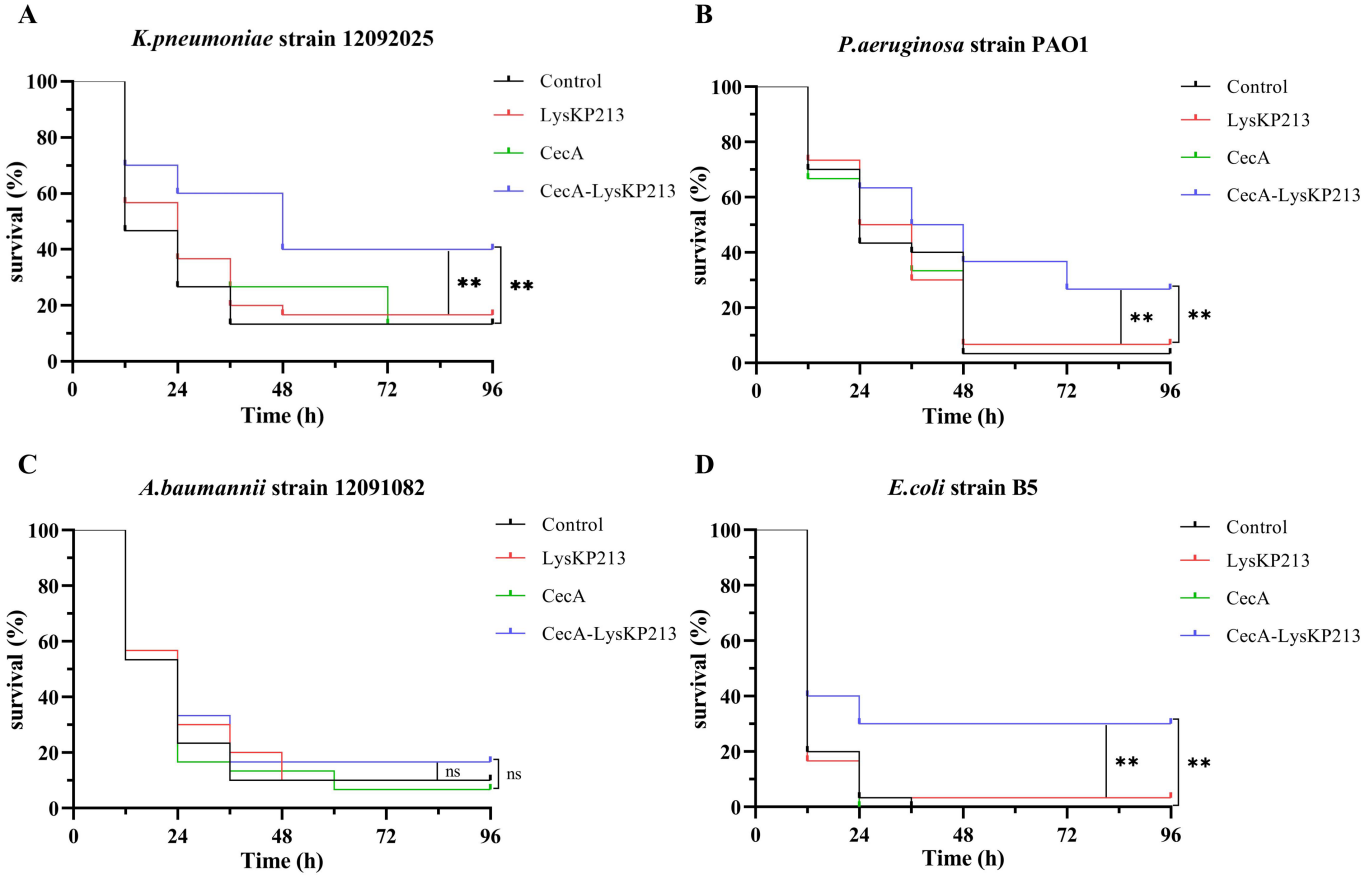
*In vivo* antibacterial activity of the engineered fusion protein CecA-LysKP213. Survival curves for *Galleria mellonella* larvae infected with *Klebsiella pneumoniae* strain 12092025 **(A)**, *Pseudomonas aeruginosa* strain PAO1 **(B)**, *Acinetobacter baumannii* strain 12091082 **(C)**, and *Escherichia coli* strain B5 **(D)**. *G. mellonella* larvae were separately infected with each of the four bacterial strains and then treated with PBS (control), CecA-LysKP213 (21 μg/mL), CecA (3 μg/mL), or LysKP213 (21 μg/mL). Larval survival was monitored after 96 h of incubation at 37°C (*n* = 10 per group). The experiment was repeated three times. **p < 0.01, n.s., not significant.

We further evaluated the antibacterial activity of CecA-LysKP213 *in vivo* using a *G. mellonella* larvae infection model with *K. pneumoniae* strain 12092025, *P. aeruginosa* strain PAO1, *A. baumannii* strain 12091082, and *E. coli* strain B5 (Figure 13). Only 10% of *G. mellonella* larvae infected with *K. pneumoniae* survived without CecA-LysKP213 treatment, whereas approximately 40% of the infected larvae survived after CecA-LysKP213 treatment (Figure 13A). Only 2% of *G. mellonella* larvae infected with *P. aeruginosa* strain PAO1 survived without CecA-LysKP213 treatment; however, approximately 30% of the infected larvae survived after CecA-LysKP213 treatment (Figure 13B). Only 10% of infected *G. mellonella* larvae infected with *A. baumannii* strain 12091082 survived without CecA-LysKP213 treatment; in contrast, approximately 16% of the infected larvae survived following CecA-LysKP213 treatment (Figure 13C). Finally, only 1% of *G. mellonella* larvae infected with *E. coli* strain B5 survived without CecA-LysKP213 treatment, while approximately 30% of the infected worms survived when after CecA-LysKP213 treatment (Figure 13D). Together, these results revealed that the *in vivo* antibacterial activity of endolysin LysKP213 against Gram-negative bacteria was improved with the addition of CecA.

## Discussion

Bacteriophages and bacteriophage-encoded endolysins display marked antibacterial activity. Moreover, bacteriophage-encoded endolysins specifically degrade the peptidoglycans in the bacterial cell wall and have low resistance potential. Nevertheless, most bacteriophage-encoded endolysins cannot easily reach the cell wall of Gram-negative bacteria owing to the presence of an OM. Consequently, different strategies have been employed aiming to solve this problem, including disrupting the OM with OM permeabilizers, such as EDTA (a chelating agent), organic acids, and colistin, or engineering endolysins such as Artilysin and CecA::ST01 (Gerstmans et al., 2016; Lim et al., 2022; Carratala et al., 2023; Zheng and Zhang, 2024). Thus, although bacteriophage-encoded endolysin therapy currently has some technology, regulatory, and logistical hurdles, the future looks promising with the emergence of engineered endolysins.

In this study, we isolated and characterized endolysin LysKP213 from *K. pneumoniae* bacteriophage KP2025. LysKP213 was found to be a lysozyme-like protein harboring a T4-like_lys domain. Recently, P9ly, which also contains a conserved T4-like_lys domain, was reported to exert antibacterial activity against different strains of bacteria, including MDR Gram-negative *Shigella dysenteriae* and Gram-positive *S. aureus* (Wang et al., 2022). Here, we evaluated the antibacterial activity spectrum of LysKP213 after EDTA treatment and observed that it possesses antibacterial activity against Gram-negative pathogens, including *K. pneumoniae*, *P. aeruginosa*, *A. baumannii*, and *E. coli*, but not against *S. aureus*, a Gram-positive bacterium.

We further found that LysKP213 retains approximately 44.4% of its lytic activity after 20 h of incubation at 95°C and approximately 57.5% residual activity after 30 min at 121°C. This indicates that LysKP213 is a highly thermostable phage-encoded endolysin. The lytic activity for most phage endolysin is within 4–40°C, which limits their application, including vaccine design, in food processing, and biotechnological production (Liu et al., 2023b). By contrast, endolysins have good (thermo)stability properties and can have a broader range of applications in human, plant/veterinary, and environmental health (Żebrowska et al., 2022; Choi and Kong, 2023; Liu et al., 2023b; Shin et al., 2023; Golosova et al., 2024). Thus, LysKP213, as a highly thermostable phage-encoded endolysin, could become useful to combat Gram-negative pathogens in the agricultural, food, and medical industries. In addition, an open question remains: what are the mechanisms responsible for the high thermal stability of LysKP213? PHAb10, a hyper-thermostable lysozyme, retains almost 100% bactericidal activity after treatment at 100°C for 1 h (Zhang et al., 2024). Here, we found that LysKP213 was similar to PHAb10 and might share similar catalytic mechanisms of action. Thus, we speculated that LysKP213 and PHAb10 might share a similar mechanism of action responsible for the high thermal stability. However, the thermal stability of phage endolysin seems to be a complex phenomenon that can be affected by many factors. Further studies are necessary to verify the mechanisms responsible for the high thermal stability of LysKP213.

Colistin (polymyxin E) and polymyxin B, the only polymyxins available commercially, have similar mechanisms of action, antimicrobial spectra, clinical uses, and toxicity. However, they also differ in several aspects, including chemical structure, formulation, potency, dosage, and pharmacokinetic properties (Kwa et al., 2007). These two medications disrupt the OM of Gram-negative bacteria via electrostatic interactions with lipopolysaccharides and phospholipids present in the OM (Kwa et al., 2007). Recently, several studies revealed that colistin in combination with phage endolysins improved antimicrobial activity against Gram-negative pathogens relative to either treatment alone (Thummeepak et al., 2016; Blasco et al., 2020; Sitthisak et al., 2023). Furthermore, one study indicated that the combined effect of polymyxin B in combination with phage endolysin, despite the absence of a synergistic effect on the inhibitory concentration, exerted a synergistic antibacterial effect against Gram-negative pathogens, including *K. pneumoniae* and *Salmonella enterica* serovar Typhimurium (Gontijo et al., 2024).

Accordingly, in this study, we further used the cationic antibiotic polymyxin B in combination with LysKP213 to overcome the barrier represented by the OM of Gram-negative bacteria. Similar to that reported for the cationic antibiotic colistin in combination with phage endolysin, the combination of LysKP213 with polymyxin B enhanced the antibacterial activity of the former against Gram-negative pathogens, including *K. pneumoniae*, *P. aeruginosa*, *A. baumannii*, and *E. coli*, both *in vitro* and *in vivo*.

AMPs with membrane-disrupting ability are found in all kingdoms of life and show promise as agents for the control of antimicrobial resistance (Zhang et al., 2021). CecA was among the first cecropins discovered in *Hyalophora cecropia* and exhibits marked antibacterial activity against Gram-negative pathogens (Moore et al., 1996; Brady et al., 2019). Several studies have reported that fusing CecA, an OM permeabilizing peptide, to the N-terminus of phage endolysins increases the antibacterial activity of the latter (Abdelkader et al., 2022; Hong et al., 2022; Lim et al., 2022). However, it was unknown whether the fusion of CecA to a phage endolysin harboring a T4-like_lys domain exerted similarly enhanced antibacterial activity. Here, we found that an engineered CecA-LysKP213 fusion protein improved the antibacterial activity of this endolysin against *K. pneumoniae*, *P. aeruginosa*, *A. baumannii*, and *E. coli in vitro* as well as *in vivo*.

Over the years, murine models have been regarded as the gold standard for studying microbial infections (Gunatilake, 2018; Hugenholtz and de Vos, 2018). However, because they have animal protection acts, ethical concerns and other drawbacks include the expensive feed costs and the special biosafety conditions. In recent years, *G. mellonella* larvae have been an alternative model to study microbial infections, including the use of *G. mellonella* larvae to evaluate the antimicrobial activity of phage and phage lysins *in vivo* (Ramarao et al., 2012; Tsai et al., 2016; Kim et al., 2018; Feng et al., 2023). The main advantage of the *G. mellonella* larval is that although it lacks an adaptive immune system, its innate immune system is remarkably similar to that of the murine innate immune system in function and structure. Secondly, *G. mellonella* larvae can survive at 37°C, equivalent to the temperature in murine. Thirdly, *G. mellonella* larvae have no ethical constraints, are cheaper to feed and breed, are easy and safe to manipulate, and have a short life cycle. Thus, the *G. mellonella* larvae *in vivo* models have been used to examine the antimicrobial activity of LysKP213 and CecA-LysKP213 and were compared with the turbidity reduction assay and CFU reduction analysis *in vitro*.

In summary, in this study, we isolated and identified endolysin LysKP213 from the *K. pneumoniae* bacteriophage KP2025. When combined with polymyxin B or fused with CecA, LysKP213 displayed enhanced antibacterial activity against *K. pneumoniae*, *P. aeruginosa*, *A. baumannii*, and *E. coli* compared to treatment with either polymyxin B alone or CecA alone, both *in vitro* and *in vivo*. These results highlight the potential of endolysin LysKP213 as a candidate antibacterial agent for the control of infections with Gram-negative pathogens.

## Data Availability

The datasets presented in this study can be found in online repositories. The names of the repository/repositories and accession number(s) can be found below: https://www.ncbi.nlm.nih.gov/genbank/, PP919962.

## Footnotes

^1^ https://rast.nmpdr.org/

^2^ https://blast.ncbi.nlm.nih.gov/Blast.cgi

^3^ https://proksee.ca

^4^ http://lowelab.ucsc.edu/cgi-bin/tRNAscan-SE2.cgi

^5^ https://cge.cbs.dtu.dk/services/VirulenceFinder/

^6^ https://cge.cbs.dtu.dk/services/ResFinder/

^7^ https://www.ebi.ac.uk/jdispatcher/

^8^ https://espript.ibcp.fr

## Supplementary material

Supplementary Figure S1 Purification of the fusion protein CecA-LysKP213. IPTG (final concentration 0.5 mM) was used to induce the overexpression of the fusion protein CecA-LysKP213; the final purified fusion protein CecA-LysKP213 was analyzed using 12% SDS-PAGE.
