# Supplementary figures and images for: The antibacterial activity of a novel highly thermostable endolysin, LysKP213, against Gram-negative pathogens is enhanced when combined with outer membrane permeabilizing agents
Source: Front Microbiol. 2024 Oct 8;15:1454618. doi: 10.3389/fmicb.2024.1454618 (PMC11493673; doi:10.3389/fmicb.2024.1454618)

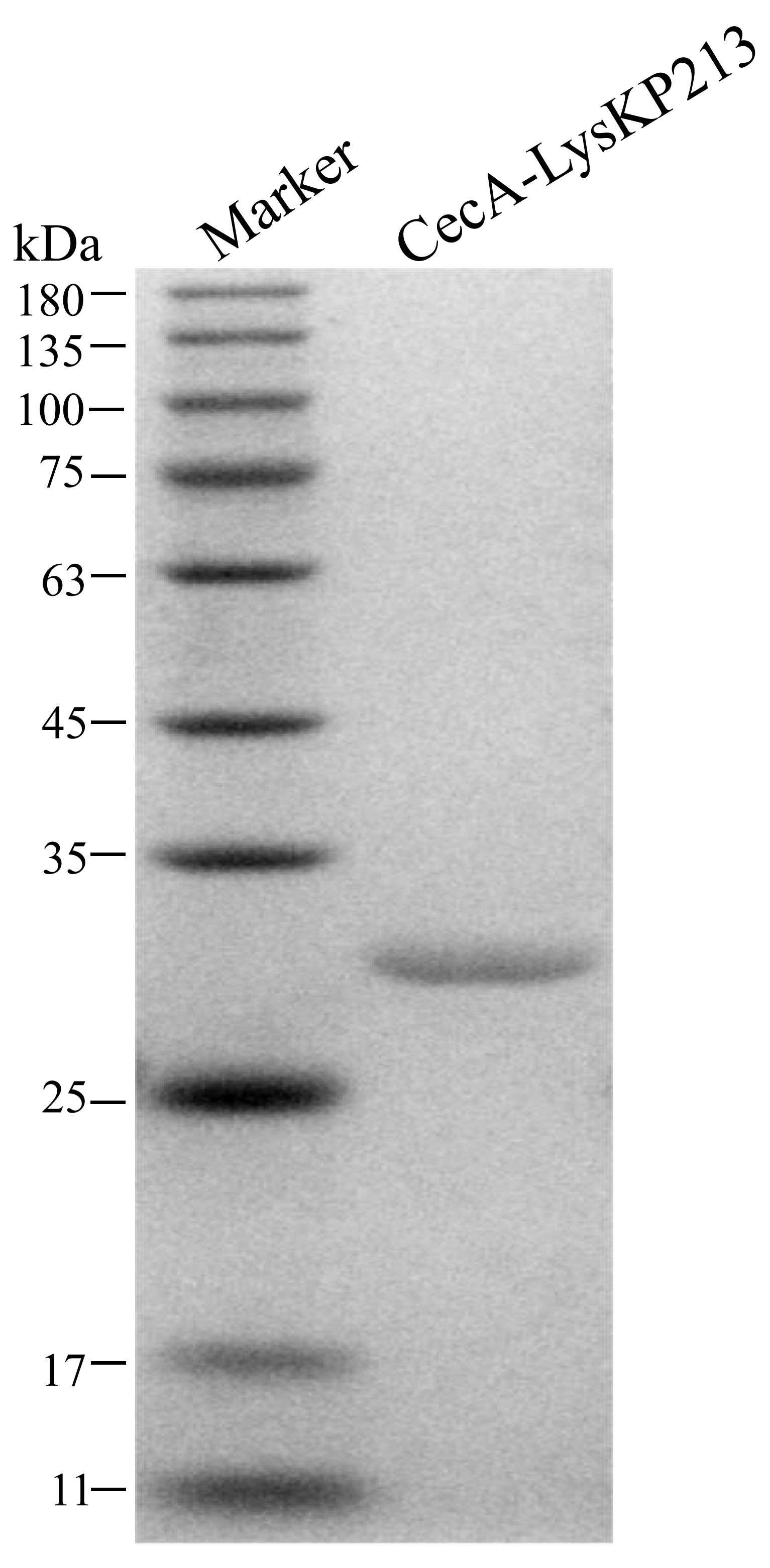

Supplement: Supplementary Figure S1 — Purification of the fusion protein CecA-LysKP213. IPTG (final concentration 0.5 mM) was used to induce the overexpression of the fusion protein CecA-LysKP213; the final purified fusion protein CecA-LysKP213 was analyzed using 12% SDS-PAGE. [file Image_1.TIF]
