# Supplementary material for: The antibacterial activity of a novel highly thermostable endolysin, LysKP213, against Gram-negative pathogens is enhanced when combined with outer membrane permeabilizing agents
Source: Front Microbiol. 2024 Oct 8;15:1454618. doi: 10.3389/fmicb.2024.1454618 (PMC11493673; doi:10.3389/fmicb.2024.1454618)
Supplement: Supplementary file 2 [file Table_1.DOCX]

**Table S1 Host range of the phage KP2025**

| **Bacterial Species** | **Strain** | **Source ^a^** | **Lysis performance ^b^** |
| --- | --- | --- | --- |
| *Klebsiella pneumoniae* | t2019100230 | C | + |
| *Klebsiella pneumoniae* | t2019101537 | C | + |
| *Klebsiella pneumoniae* | t2019101617 | C | + |
| *Klebsiella pneumoniae* | n2021021901 | C | + |
| *Klebsiella pneumoniae* | w2021060915 | C | + |
| *Klebsiella pneumoniae* | t2021060818 | C | + |
| *Klebsiella pneumoniae* | 201909376 | C | + |
| *Klebsiella pneumoniae* | 201908971 | C | + |
| *Klebsiella pneumoniae* | 12102041 | C | + |
| *Klebsiella pneumoniae* | 12092025 | C | + |
| *Klebsiella pneumoniae* | 12122012 | C | + |
| *Klebsiella pneumoniae* | 11112046 | C | + |
| *Klebsiella pneumoniae* | 11151006 | C | + |
| *Klebsiella pneumoniae* | 12104027 | C | + |
| *Klebsiella pneumoniae* | 11272018 | C | + |
| *Klebsiella pneumoniae* | 11272077 | C | + |
| *Klebsiella pneumoniae* | 505257 | A | + |
| *Klebsiella pneumoniae* | CRKP-5 | C | + |
| *Klebsiella pneumoniae* | CRKP-2 | C | + |
| *Klebsiella pneumoniae* | CRKP-3 | C | + |
| *Klebsiella pneumoniae* | HZY14 | C | + |
| *Klebsiella pneumoniae* | HZY10 | C | + |
| *Klebsiella pneumoniae* | 25102 | A | + |
| *Klebsiella pneumoniae* | 29260 | C | + |
| *Klebsiella pneumoniae* | 361-39 | B | + |
| *Klebsiella pneumoniae* | 30826 | A | + |
| *Klebsiella pneumoniae* | C4 | B | + |
| *Escherichia coli* | n2020062903 | C | + |
| *Escherichia coli* | w2019102206 | C | + |
| *Escherichia coli* | w2019100204 | C | + |
| *Escherichia coli* | n2019090303 | C | + |

^a^ A: Zhu jiang Hospital of Southern Medical University; B: Fudan University; C: Kai Yang People Hospital.

^b^ +: Lytic plaques were observed.
