# Supplementary material for: The antibacterial activity of a novel highly thermostable endolysin, LysKP213, against Gram-negative pathogens is enhanced when combined with outer membrane permeabilizing agents
Source: Front Microbiol. 2024 Oct 8;15:1454618. doi: 10.3389/fmicb.2024.1454618 (PMC11493673; doi:10.3389/fmicb.2024.1454618)
Supplement: Supplementary file 3 [file Table_2.DOCX]

**Table S2 Predicted functions of phage KP2025**

| **ORF#** | **Strand** | **Nucleotide position** | **Best annotated protein** |
| --- | --- | --- | --- |
| ORF1 | + | 98-1669 | portal protein |
| ORF2 | + | 1755-2234 | serine protease |
| ORF3 | + | 2237-3364 | putative scaffold protein |
| ORF4 | + | 3383-3775 | Head decoration protein |
| ORF5 | + | 3800-4801 | major capsid protein |
| ORF6 | + | 4881-5240 | hypothetical protein |
| ORF7 | + | 5250-5798 | putative tail capping protein |
| ORF8 | + | 5798-6286 | virion morphogenesis protein |
| ORF9 | + | 6332-6748 | Minor capsid protein |
| ORF10 | + | 6741-7406 | TC1 tail completion protein |
| ORF11 | + | 7484-8890 | tail sheath protein |
| ORF12 | + | 8939-9418 | protein of unknown function (DUF3277) |
| ORF13 | + | 9461-9940 | tail assembly chaperone protein |
| ORF14 | + | 9982-10215 | hypothetical protein |
| ORF15 | + | 10228-12201 | hypothetical protein |
| ORF16 | + | 12254-12934 | hypothetical protein |
| ORF17 | + | 12944-13363 | hypothetical protein |
| ORF18 | + | 13378-14379 | tail protein |
| ORF19 | + | 14381-15079 | Baseplate protein |
| ORF20 | + | 15081-15701 | Baseplate wedge protein |
| ORF21 | + | 15711-15965 | hypothetical protein |
| ORF22 | + | 15958-17442 | Baseplate wedge protein |
| ORF23 | + | 17446-18072 | hypothetical protein |
| ORF24 | + | 18082-19122 | potential tail fiber or connector protein |
| ORF25 | + | 19124-19597 | hypothetical protein |
| ORF26 | + | 19616-19960 | hypothetical protein |
| ORF27 | + | 19960-22797 | tail spike protein |
| ORF28 | + | 22868-23938 | hypothetical protein |
| ORF29 | + | 23952-26381 | putative tail fiber protein |
| ORF30 | - | 27353-26475 | Single-stranded DNA-binding protein |
| ORF31 | - | 27767-27369 | hypothetical protein |
| ORF32 | - | 28535-27819 | hypothetical protein |
| ORF33 | - | 29138-28548 | AP2 domain-containing protein |
| ORF34 | - | 29939-29166 | DNA polymerase |
| ORF35 | - | 32147-30417 | DNA polymerase |
| ORF36 | - | 32477-32160 | hypothetical protein |
| ORF37 | - | 34286-32517 | DNA directed DNA polymerase |
| ORF38 | - | 34562-34299 | hypothetical protein |
| ORF39 | - | 35536-34574 | putative disaccharide synthase |
| ORF40 | - | 36116-35547 | putative glycosyltransferase |
| ORF41 | - | 36377-36180 | hypothetical protein |
| ORF42 | - | 36579-36355 | hypothetical protein |
| **ORF#** | **Strand** | **Nucleotide position** | **Best annotated protein** |
| ORF43 | - | 36860-36579 | hypothetical protein |
| ORF44 | - | 37384-36869 | hypothetical protein |
| ORF45 | - | 38096-37470 | hypothetical protein |
| ORF46 | - | 38758-38156 | TerD-like protein |
| ORF47 | - | 39648-38854 | VWA domain-containing protein |
| ORF48 | - | 39806-39645 | Homeodomain leucine-zipper |
| ORF49 | - | 40212-39799 | hypothetical protein |
| ORF50 | - | 41319-40222 | Toxic anion resistance protein |
| ORF51 | - | 41907-41329 | Spr T-like family |
| ORF52 | - | 42452-41910 | hypothetical protein |
| ORF53 | - | 42762-42523 | hypothetical protein |
| ORF54 | - | 43338-42775 | hypothetical protein |
| ORF55 | - | 43531-43325 | hypothetical protein |
| ORF56 | - | 43707-43528 | hypothetical protein |
| ORF57 | - | 44729-43749 | Integral membrane protein |
| ORF58 | - | 45052-44810 | hypothetical protein |
| ORF59 | - | 45389-45177 | hypothetical protein |
| ORF60 | - | 45859-45398 | macro domain-containing protein |
| ORF61 | - | 46705-45869 | hypothetical protein |
| ORF62 | - | 47357-46698 | putative hydrolase |
| ORF63 | - | 47964-47359 | hypothetical protein |
| ORF64 | - | 48166-47975 | hypothetical protein |
| ORF65 | - | 49377-48217 | Bacterial DNA protein |
| ORF66 | - | 50410-49445 | hypothetical protein |
| ORF67 | - | 50684-50388 | hypothetical protein |
| ORF68 | - | 51115-50681 | Yqe Y-like protein |
| ORF69 | - | 51417-51112 | hypothetical protein |
| ORF70 | - | 51764-51417 | hypothetical protein |
| ORF71 | - | 51993-51805 | hypothetical protein |
| ORF72 | - | 54872-51993 | putative DNA helicase |
| ORF73 | - | 55359-54865 | hypothetical protein |
| ORF74 | - | 55878-55369 | hypothetical protein |
| ORF75 | - | 56260-55895 | hypothetical protein |
| ORF76 | - | 56522-56271 | protein of unknown function（DUF4326） |
| ORF77 | - | 56896-56522 | protein of unknown function（DUF4326） |
| ORF78 | - | 57255-56893 | hypothetical protein |
| ORF79 | - | 57533-57252 | hypothetical protein |
| ORF80 | - | 57754-57530 | hypothetical protein |
| ORF81 | - | 57908-57771 | hypothetical protein |
| ORF82 | - | 58582-57887 | Nicotinamide mononucleotide transporter |
| ORF83 | - | 58915-58601 | hypothetical protein |
| ORF84 | - | 59098-58925 | hypothetical protein |
| ORF85 | - | 59333-59109 | hypothetical protein |
| **ORF#** | **Strand** | **Nucleotide position** | **Best annotated protein** |
| ORF86 | - | 60402-59335 | trifunctional NAD biosynthesis |
| ORF87 | - | 60872-60420 | hypothetical protein |
| ORF88 | - | 61082-60882 | hypothetical protein |
| ORF89 | - | 61649-61374 | hypothetical protein |
| ORF90 | - | 61939-61661 | hypothetical protein |
| ORF91 | - | 62372-61944 | hypothetical protein |
| ORF92 | - | 62519-62382 | hypothetical protein |
| ORF93 | - | 62845-62519 | hypothetical protein |
| ORF94 | - | 63222-62845 | hypothetical protein |
| ORF95 | - | 63388-63224 | BNR/Asp-box repeat protein |
| ORF96 | - | 63570-63391 | hypothetical protein |
| ORF97 | - | 64004-63567 | hypothetical protein |
| ORF98 | - | 64372-64004 | hypothetical protein |
| ORF99 | + | 64489-64719 | hypothetical protein |
| ORF100 | + | 64722-65015 | hypothetical protein |
| ORF101 | + | 65029-65499 | hypothetical protein |
| ORF102 | + | 65504-65896 | hypothetical protein |
| ORF103 | + | 65906-66241 | hypothetical protein |
| ORF104 | + | 66241-66483 | hypothetical protein |
| ORF105 | + | 66480-66731 | hypothetical protein |
| ORF106 | + | 66746-67138 | hypothetical protein |
| ORF107 | + | 67348-68472 | replicative helicase |
| ORF108 | + | 68514-68861 | hypothetical protein |
| ORF109 | + | 68858-69475 | hypothetical protein |
| ORF110 | - | 70476-70282 | hypothetical protein |
| ORF111 | + | 70663-70884 | hypothetical protein |
| ORF112 | + | 70974-71510 | hypothetical protein |
| ORF113 | + | 71573-71863 | DNA ligase |
| ORF114 | + | 71945-72091 | portal protein |
| ORF115 | + | 72132-72359 | hypothetical protein |
| ORF116 | + | 72363-72911 | hypothetical protein |
| ORF117 | + | 72991-73107 | hypothetical protein |
| ORF118 | + | 73197-73424 | hypothetical protein |
| ORF119 | + | 73527-73688 | hypothetical protein |
| ORF120 | + | 73782-74027 | hypothetical protein |
| ORF121 | + | 74109-74282 | hypothetical protein |
| ORF122 | + | 74363-74584 | Radical S-adenosyl methionine containing protein |
| ORF123 | + | 74633-74860 | hypothetical protein |
| ORF124 | + | 74948-75100 | hypothetical protein |
| ORF125 | + | 75112-75468 | hypothetical protein |
| ORF126 | + | 75561-75932 | hypothetical protein |
| ORF127 | + | 76009-76143 | hypothetical protein |
| ORF128 | + | 76261-76443 | hypothetical protein |
| **ORF#** | **Strand** | **Nucleotide position** | **Best annotated protein** |
| ORF129 | + | 76535-76918 | hypothetical protein |
| ORF130 | + | 76974-77249 | hypothetical protein |
| ORF131 | + | 77508-78074 | hypothetical protein |
| ORF132 | + | 78162-78386 | hypothetical protein |
| ORF133 | + | 78448-78684 | hypothetical protein |
| ORF134 | + | 78778-79077 | hypothetical protein |
| ORF135 | + | 79118-79381 | hypothetical protein |
| ORF136 | + | 79395-79688 | L20 Mitochondrial ribosomal protein subunit L20 |
| ORF137 | + | 79764-79961 | hypothetical protein |
| ORF138 | + | 79973-80356 | hypothetical protein |
| ORF139 | + | 80442-80705 | hypothetical protein |
| ORF140 | + | 80752-80946 | hypothetical protein |
| ORF141 | + | 80948-81274 | hypothetical protein |
| ORF142 | + | 81739-82107 | hypothetical protein |
| ORF143 | - | 82861-82742 | hypothetical protein |
| ORF144 | + | 83036-83167 | hypothetical protein |
| ORF145 | + | 83781-83924 | hypothetical protein |
| ORF146 | + | 83974-84285 | type AsnC-type helix-turn-helix domain |
| ORF147 | + | 84318-84515 | hypothetical protein |
| ORF148 | + | 84521-84685 | hypothetical protein |
| ORF149 | + | 84682-85392 | hypothetical protein |
| ORF150 | + | 85389-85754 | DUF551 domain-containing protein |
| ORF151 | + | 85945-86448 | hypothetical protein |
| ORF152 | + | 86457-86696 | hypothetical protein |
| ORF153 | + | 86693-86923 | hypothetical protein |
| ORF154 | + | 86935-87120 | putative integrase |
| ORF155 | + | 87131-87301 | hypothetical protein |
| ORF156 | + | 87382-87672 | hypothetical protein |
| ORF157 | + | 87733-87900 | cytochrome C-like protein |
| ORF158 | + | 87900-89108 | hypothetical protein |
| ORF159 | + | 89105-89431 | hypothetical protein |
| ORF160 | + | 89433-90011 | hypothetical protein |
| ORF161 | + | 90004-90261 | hypothetical protein |
| ORF162 | + | 90258-90479 | Flagellar and Swarming motility protein |
| ORF163 | + | 90469-90951 | hypothetical protein |
| ORF164 | + | 90961-91116 | hypothetical protein |
| ORF165 | + | 91181-91333 | hypothetical protein |
| ORF166 | + | 91362-91625 | hypothetical protein |
| ORF167 | + | 91634-91876 | hypothetical protein |
| ORF168 | + | 91902-92192 | hypothetical protein |
| ORF169 | + | 92203-92631 | hypothetical protein |
| ORF170 | + | 92842-93075 | hypothetical protein |
| ORF171 | + | 93169-93360 | hypothetical protein |
| **ORF#** | **Strand** | **Nucleotide position** | **Best annotated protein** |
| ORF172 | + | 93357-93764 | hypothetical protein |
| ORF173 | + | 93748-94032 | L1 transposable element dsRBD-like domain |
| ORF174 | + | 94029-94187 | hypothetical protein |
| ORF175 | + | 94204-94836 | glycosyltransferase |
| ORF176 | + | 94847-95056 | hypothetical protein |
| ORF177 | + | 95058-95423 | hypothetical protein |
| ORF178 | + | 95420-96043 | hypothetical protein |
| ORF179 | + | 96046-96168 | hypothetical protein |
| ORF180 | + | 96224-96397 | hypothetical protein |
| ORF181 | + | 96407-96529 | hypothetical protein |
| ORF182 | + | 96526-96909 | hypothetical protein |
| ORF183 | + | 96911-97201 | hypothetical protein |
| ORF184 | + | 97210-97962 | hypothetical protein |
| ORF185 | + | 98008-98232 | CcmH protein |
| ORF186 | + | 98219-98443 | hypothetical protein |
| ORF187 | - | 99472-98657 | NAD-dependent protein deacetylase of SIR2 |
| ORF188 | - | 100248-99589 | hypothetical protein |
| ORF189 | - | 100945-100217 | DNA cytosine methyltransferase |
| ORF190 | - | 101334-100945 | hypothetical protein |
| ORF191 | - | 102270-101335 | Protein HNS-Dependent expression a helical |
| ORF192 | - | 102743-102378 | hypothetical protein |
| ORF193 | - | 103140-102946 | hypothetical protein |
| ORF194 | - | 103593-103156 | hypothetical protein |
| ORF195 | - | 104023-103604 | hypothetical protein |
| ORF196 | - | 104297-104013 | hypothetical protein |
| ORF197 | - | 104471-104313 | hypothetical protein |
| ORF198 | - | 104938-104495 | hypothetical protein |
| ORF199 | - | 105456-104938 | hypothetical protein |
| ORF200 | - | 106021-105449 | YspA |
| ORF201 | - | 106306-106031 | hypothetical protein |
| ORF202 | - | 107043-106306 | threonine-protein phosphatase 2 |
| ORF203 | - | 107625-107053 | putative aminoacyl-tRNA synthetase |
| ORF204 | - | 107832-107635 | hypothetical protein |
| ORF205 | - | 108503-107841 | integrase |
| ORF206 | - | 108756-108553 | hypothetical protein |
| ORF207 | - | 108989-108765 | hypothetical protein |
| ORF208 | - | 109509-109000 | adenine-specific methyltransferase |
| ORF209 | - | 109927-109493 | hypothetical protein |
| ORF210 | - | 110590-109964 | ATP-dependent protease |
| ORF211 | - | 111083-110592 | ABC transporter |
| ORF212 | - | 111877-111122 | PhoH-like protein |
| ORF213 | - | 112414-111914 | lysozyme |
| ORF214 | - | 112568-112422 | hypothetical protein |
| **ORF#** | **Strand** | **Nucleotide position** | **Best annotated protein** |
| ORF215 | - | 112839-112573 | hypothetical protein |
| ORF216 | - | 113678-112836 | dTDP-4-dehydrorhamnose reductase |
| ORF217 | - | 114244-113675 | dTDP-4-dehydrorhamnose 3, 5-epimerase |
| ORF218 | - | 114582-114256 | hypothetical protein |
| ORF219 | - | 114785-114582 | hypothetical protein |
| ORF220 | - | 115880-114795 | Ribonucleotide reductase of class beta subunit |
| ORF221 | - | 116209-115880 | hypothetical protein |
| ORF222 | - | 118500-116251 | Ribonucleotide reductase of class alpha subunit |
| ORF223 | - | 118759-118511 | hypothetical protein |
| ORF224 | - | 119185-118772 | hypothetical protein |
| ORF225 | - | 120242-119274 | putative thymidylate synthase |
| ORF226 | - | 120851-120252 | hypothetical protein |
| ORF227 | - | 121466-120894 | HNH endonuclease |
| ORF228 | - | 121647-121459 | hypothetical protein |
| ORF229 | - | 122032-121634 | hypothetical protein |
| ORF230 | - | 123041-122073 | DNA polymerase B |
| ORF231 | - | 123727-123038 | Exodeoxyribonuclease |
| ORF232 | - | 124372-124163 | Gram-negative pili assembly chaperone |
| ORF233 | - | 125331-124447 | DNA recombination-dependent growth factor C |
| ORF234 | - | 125625-125341 | hypothetical protein |
| ORF235 | - | 126385-125663 | hydrolase |
| ORF236 | - | 126574-126395 | hypothetical protein |
| ORF237 | - | 127702-126584 | adenylation DNA ligase-like protein |
| ORF238 | - | 127936-127712 | hypothetical protein |
| ORF239 | - | 128118-127933 | zinc knuckle protein |
| ORF240 | - | 128357-128115 | hypothetical protein |
| ORF241 | - | 128518-128360 | hypothetical protein |
| ORF242 | - | 128934-128530 | ATP-binding protein |
| ORF243 | - | 129845-128931 | RNA ligase |
| ORF244 | - | 130057-129842 | hypothetical protein |
| ORF245 | - | 130607-130059 | hypothetical protein |
| ORF246 | - | 131100-130609 | hypothetical protein |
| ORF247 | - | 131453-131100 | hypothetical protein |
| ORF248 | - | 131619-131440 | hypothetical protein |
| ORF249 | - | 131783-131616 | hypothetical protein |
| ORF250 | - | 131929-131798 | hypothetical protein |
| ORF251 | - | 132158-131943 | hypothetical protein |
| ORF252 | - | 132510-132169 | hypothetical protein |
| ORF253 | - | 132662-132507 | hypothetical protein |
| ORF254 | - | 132985-132659 | putative transposase-like protein |
| ORF255 | - | 133360-133175 | hypothetical protein |
| ORF256 | - | 133580-133362 | hypothetical protein |
| ORF257 | - | 133882-133580 | hypothetical protein |
| **ORF#** | **Strand** | **Nucleotide position** | **Best annotated protein** |
| ORF258 | - | 134041-133901 | hypothetical protein |
| ORF259 | + | 134319-134531 | hypothetical protein |
| ORF260 | + | 134521-135414 | Baseplate wedge protein |
| ORF261 | + | 135425-135628 | hypothetical protein |
| ORF262 | + | 135700-137418 | Nicotinamide phosphoribosyl transferase |
| ORF263 | + | 137497-137718 | TFIIB Transcription factor zinc-finger |
| ORF264 | + | 137771-138046 | hypothetical protein |
| ORF265 | + | 138191-138655 | hypothetical protein |
| tRNA1 | + | 138657-138737 | tRNA-Leu-TAA |
| ORF266 | + | 138830-138964 | hypothetical protein |
| tRNA2 | + | 139048-139119 | tRNA-Cys-GCA |
| tRNA3 | + | 139694-139765 | tRNA-Sup-CTA |
| ORF267 | + | 139786-139902 | hypothetical protein |
| ORF268 | + | 139886-140071 | hypothetical protein |
| tRNA4 | + | 140087-140518 | tRNA-Arg-TCT |
| ORF269 | + | 140183-141613 | protein of unknown function (DUF2828) |
| tRNA5 | + | 142312-142398 | tRNA-Pseudo-TGA |
| tRNA6 | + | 142494-142576 | tRNA-Ser-GCT |
| tRNA7 | + | 142688-142761 | tRNA-Lys-TTT |
| tRNA8 | + | 142771-142851 | tRNA-Tyr-GTA |
| tRNA9 | + | 143045-143126 | tRNA-Asn-GTT |
| tRNA10 | + | 143134-143206 | tRNA-Thr-TGT |
| tRNA11 | + | 143451-143521 | tRNA-Gly-TCC |
| tRNA12 | + | 143740-143812 | tRNA-Gln-TTG |
| tRNA13 | + | 143901-143975 | tRNA-Pro-TGG |
| tRNA14 | + | 144119-144190 | tRNA-Phe-GAA |
| tRNA15 | + | 144323-144395 | tRNA-Arg-ACG |
| tRNA16 | + | 144735-144818 | tRNA-Leu-TAG |
| tRNA17 | + | 145058-145129 | tRNA-Val-TAC |
| ORF270 | + | 145220-145483 | hypothetical protein |
| tRNA18 | + | 145573-145646 | tRNA-Met-CAT |
| tRNA19 | + | 145859-145931 | tRNA-Ile-GAT |
| ORF271 | + | 146105-146392 | hypothetical protein |
| ORF272 | + | 146555-146728 | hypothetical protein |
| ORF273 | + | 146761-147195 | hypothetical protein |
| ORF274 | + | 147272-147430 | hypothetical protein |
| ORF275 | + | 147439-149523 | Large subunit terminase |
