# Supplementary material for: The antibacterial activity of a novel highly thermostable endolysin, LysKP213, against Gram-negative pathogens is enhanced when combined with outer membrane permeabilizing agents
Source: Front Microbiol. 2024 Oct 8;15:1454618. doi: 10.3389/fmicb.2024.1454618 (PMC11493673; doi:10.3389/fmicb.2024.1454618)
Supplement: Supplementary file 4 [file Table_3.DOCX]

**Table S3 T4L-like lysozymes with characterized structures**

| **Protein** | **Catalytic Residues** | **PDB ID** |
| --- | --- | --- |
| LysKP213 | Glu15 Asp24 Thr30 | 7RGR |
| PHAb10 | Glu17 Asp26 Thr32 | 7YKU |
| PHAb8 | Glu74 Asp83 Thr89 | 8HEM |
| LysF1 | Glu15 Asp24 Thr30 | 7M5I |
| T4L | Glu11 Asp20 Thr26 | 1LYD |
