# Supplementary material for: The antibacterial activity of a novel highly thermostable endolysin, LysKP213, against Gram-negative pathogens is enhanced when combined with outer membrane permeabilizing agents
Source: Front Microbiol. 2024 Oct 8;15:1454618. doi: 10.3389/fmicb.2024.1454618 (PMC11493673; doi:10.3389/fmicb.2024.1454618)
Supplement: Supplementary file 5 [file Table_4.DOCX]

**Table S4 Test strains for LysKP213 Lytic activity spectrum**

| **Bacterial Species** | **Strain** | **Source ^a^** |
| --- | --- | --- |
| *Klebsiella pneumoniae* | C1 | A |
| *Klebsiella pneumoniae* | C2 | A |
| *Klebsiella pneumoniae* | C3 | A |
| *Klebsiella pneumoniae* | C6 | A |
| *Klebsiella pneumoniae* | C7 | A |
| *Klebsiella pneumoniae* | C8 | A |
| *Klebsiella pneumoniae* | C9 | A |
| *Klebsiella pneumoniae* | C10 | A |
| *Klebsiella pneumoniae* | C11 | A |
| *Klebsiella pneumoniae* | C12 | A |
| *Klebsiella pneumoniae* | C13 | A |
| *Klebsiella pneumoniae* | C14 | A |
| *Klebsiella pneumoniae* | C15 | A |
| *Klebsiella pneumoniae* | C16 | A |
| *Klebsiella pneumoniae* | C17 | A |
| *Klebsiella pneumoniae* | 30826 | B |
| *Klebsiella pneumoniae* | CRKP-4 | C |
| *Klebsiella pneumoniae* | 11272028 | C |
| *Klebsiella pneumoniae* | 201908971 | C |
| *Acinetobacter baumannii* | E3 | A |
| *Acinetobacter baumannii* | E4 | A |
| *Acinetobacter baumannii* | E5 | A |
| *Acinetobacter baumannii* | E10 | A |
| *Acinetobacter baumannii* | E11 | A |
| *Acinetobacter baumannii* | E12 | A |
| *Acinetobacter baumannii* | 11152049 | C |
| *Acinetobacter baumannii* | 11146008 | C |
| *Acinetobacter baumannii* | 12082041 | C |
| *Acinetobacter baumannii* | 12091082 | C |
| *Acinetobacter baumannii* | 11264018 | C |
| *Acinetobacter baumannii* | 12082049 | C |
| *Acinetobacter baumannii* | AB19606 | D |
| *Acinetobacter baumannii* | t2019091102 | C |
| *Acinetobacter baumannii* | t2019091105 | C |
| *Acinetobacter baumannii* | t2019090715 | C |
| *Pseudomonas aeruginosa* | D2 | A |
| *Pseudomonas aeruginosa* | D4 | A |
| *Pseudomonas aeruginosa* | D6 | A |
| *Pseudomonas aeruginosa* | D7 | A |
| *Pseudomonas aeruginosa* | PAO1 | E |
| *Pseudomonas aeruginosa* | 201909997 | C |
| *Escherichia coli* | B1 | A |
| **Bacterial Species** | **Strain** | **Source ^a^** |
| *Escherichia coli* | B2 | A |
| *Escherichia coli* | B3 | A |
| *Escherichia coli* | B4 | A |
| *Escherichia coli* | B5 | A |
| *Escherichia coli* | B6 | A |
| *Escherichia coli* | B7 | A |
| *Escherichia coli* | B8 | A |
| *Escherichia coli* | B9 | A |
| *Escherichia coli* | B10 | A |
| *Escherichia coli* | B16 | A |
| *Escherichia coli* | B17 | A |
| *Escherichia coli* | n2019090303 | C |
| *Escherichia coli* | n2020062903 | C |
| *Escherichia coli* | w2019101601 | C |
| *Escherichia coli* | w2019101609 | C |
| *Escherichia coli* | w2019102206 | C |
| *Escherichia coli* | w2019100204 | C |
| *Staphylococcus aureus* | A1 | A |
| *Staphylococcus aureus* | A2 | A |
| *Staphylococcus aureus* | A3 | A |
| *Staphylococcus aureus* | A6 | A |
| *Staphylococcus aureus* | A7 | A |
| *Staphylococcus aureus* | A8 | A |
| *Staphylococcus aureus* | A9 | A |
| *Staphylococcus aureus* | A10 | A |
| *Staphylococcus aureus* | A11 | A |
| *Staphylococcus aureus* | A12 | A |
| *Staphylococcus aureus* | A14 | A |
| *Staphylococcus aureus* | A21 | A |
| *Staphylococcus aureus* | A26 | A |
| *Staphylococcus aureus* | A35 | A |
| *Staphylococcus aureus* | A36 | A |
| *Staphylococcus aureus* | A37 | A |
| *Staphylococcus aureus* | A38 | A |
| *Staphylococcus aureus* | A39 | A |

^a^ A: Maternal and Child Health Hospital of Guiyang City; B: Zhu jiang Hospital of Southern Medical University; C: Kai Yang People Hospital; D: China Medical culture collection; E: Army Medical University.
