# Supplementary material for: The antibacterial activity of a novel highly thermostable endolysin, LysKP213, against Gram-negative pathogens is enhanced when combined with outer membrane permeabilizing agents
Source: Front Microbiol. 2024 Oct 8;15:1454618. doi: 10.3389/fmicb.2024.1454618 (PMC11493673; doi:10.3389/fmicb.2024.1454618)
Supplement: Supplementary file 6 [file Table_5.DOCX]

**Table S5 MICs of polymyxin B (PMB)**

| **Bacterial Species** | **Strain** | **MIC_PMB_ (µg/mL)** |
| --- | --- | --- |
| *Escherichia coli* | B5 | 1 |
| *Pseudomonas aeruginosa* | PAO1 | 0.5 |
| *Klebsiella pneumoniae* | 12092025 | 1 |
| *Acinetobacter baumannii* | 12091082 | 2 |
